# Ripening-induced chemical modifications of papaya pectin inhibit cancer cell proliferation

**DOI:** 10.1038/s41598-017-16709-3

**Published:** 2017-11-29

**Authors:** Samira Bernardino Ramos do Prado, Gabrielle Fernandez Ferreira, Yosuke Harazono, Tânia Misuzu Shiga, Avraham Raz, Nicholas C. Carpita, João Paulo Fabi

**Affiliations:** 1 Department of Food Science and Experimental Nutrition, School of Pharmaceutical Sciences, University of São Paulo, São Paulo, SP Brazil; 2 Departments of Oncology and Pathology, School of Medicine, Wayne State University, and Karmanos Cancer Institute, Detroit, MI USA; 3 Department of Maxillofacial Surgery, Tokyo Medical and Dental University, Bunkyo-ku, Tokyo, 113–8510 Japan; 4 Department of Botany & Plant Pathology, Purdue University, West Lafayette, IN USA; 5 Food and Nutrition Research Center (NAPAN), University of São Paulo, São Paulo, SP Brazil; 6 Food Research Center (FoRC), CEPID-FAPESP (Research, Innovation and Dissemination Centers, São Paulo Research Foundation), São Paulo, SP Brazil

## Abstract

Papaya (*Carica papaya* L.) is a fleshy fruit with a rapid pulp softening during ripening. Ripening events are accompanied by gradual depolymerization of pectic polysaccharides, including homogalacturonans, rhamnogalacturonans, arabinogalactans, and their modified forms. During intermediate phases of papaya ripening, partial depolymerization of pectin to small size with decreased branching had enhanced pectin anti-cancer properties. These properties were lost with continued decomposition at later phases of ripening. Pectin extracted from intermediate phases of papaya ripening markedly decreased cell viability, induced necroptosis, and delayed culture wound closing in three types of immortalized cancer cell lines. The possible explanation for these observations is that papaya pectins extracted from the third day after harvesting have disrupted interaction between cancer cells and the extracellular matrix proteins, enhancing cell detachment and promoting apoptosis/necroptosis. The anticancer activity of papaya pectin is dependent on the presence and the branch of arabinogalactan type II (AGII) structure. These are first reports of AGII in papaya pulp and the first reports of an *in vitro* biological activity of papaya pectins that were modified by natural action of ripening-induced pectinolytic enzymes. Identification of the specific pectin branching structures presents a biological route to enhancing anti-cancer properties in papaya and other climacteric fruits.

## Introduction

Dietary fiber are generally considered carbohydrates that are incompletely processed by human digestive enzymes^1^, but can provide health benefits^2^, such as lowering the risk of colorectal cancer development^3^. Fruits and vegetables are rich in pectin, a soluble dietary fiber found in plant cell walls^4^. Pectin is a complex structure comprising two principal polymers of homogalacturonan (HG) and rhamnogalacturonan type I (RG-I), but each can be modified through side-croup addition to add functional complexity. HGs are linear homopolymers composed of *1*,*4*-α-D-galacturonic acid (GalA) residues in which some carboxyl groups are esterified with methyl and acetyl groups. Addition of Xyl residues forms xylogalacturonan, and addition of four highly conserved oligomers of 21 different sugar residues forms the boron-crosslinking rhamnogalacturonan type II (RG-II). RG-Is are defined by a backbone of 4-α-D-GalA-1-2-α-L-Rha-1 repeating units, with highly branched side-chains of arabinans, galactans and arabinogalactans attached to the Rha residues^5^.

Many types of pectin, especially the modified ones, have been associated with anti-cancer activity in both *in vitro* and *in vivo* studies, such as the reduction of cell proliferation, migration, adhesion, and the induction of apoptosis^6–10^. These anti-cancer activities were shown for modified pectins of citrus^11–14^, apple^15,16^, sugar beet^6^. and ginseng^8^. The biological effects of modified pectin have been associated, at least partially, with the inhibition of galectin-3 function, a multifaceted and pro-metastatic protein whose expression is up-regulated in many cancers^14,17–19^. Pectin modification decreases the overall molecular weight, thereby releasing fragments of RG-I that can bind to galectin-3^20^. HG and RG-I fragments are known to induce cancer cell detachment^7,9^, but lack of structural-functional relationships makes determination of specific anti-cancer activities difficult. Moreover, pectin from different sources can vary widely in size, composition and branching pattern^21^, and consequently, tracing anti-cancer properties to specific carbohydrate structures and interactions is still poorly understood. To our knowledge, there are no reports that have investigated the association between the alterations of pectin structure by endogenous action of pectolytic enzymes and the anti-cancer activities.

Climacteric fleshy fruits shows substantial changes in the pulp cell wall polysaccharides as they ripen^22^. Thus, physiological modification of cell wall during ripening could be an alternative to pectin modification as several cell wall degrading enzymes are coordinately expressed throughout ripening^23^. Papaya is a climacteric fleshy fruit with a fast ripening and a massive solubilization of galacturonan chains arose from extensive action of pectinolytic enzymes during ripening^24–26^. Thus, increased action of cell wall degrading enzymes during ripening of papaya and consequent decrease of pectin molecular weight^27^ might naturally modify pectin structures possibly increasing pectin’s anti-cancer activity. As such, the present study aimed to characterize and to evaluate pectin isolated from papaya fruits harvested at different ripening stages to investigate the relationship between changes in pectin’s structure and their anti-proliferative activity on three cancer cell lines.

## Results

### Papaya pectin from different ripening stages induces death of cancer cells at different levels

The three cell lines used in this work showed different responses to papaya pectin treatment based on their different types of mutations and different grades of aggressiveness. HCT116 is *KRAS* ^*G13D*^, HT29 is *BRAF* ^*V600E*^ and PC3 is *KRAS* and *BRAF* wild type and possess a mutation on p53, though^28,29^. HCT116 has an undifferentiated phenotype with a high metastatic potential and an unstable adherent junctions^30^. In turn, HT29 is differentiated with less aggressive behavior^31^. PC3 cells represent very aggressive forms of prostatic adenocarcinoma^32^.

**Figure 1:**
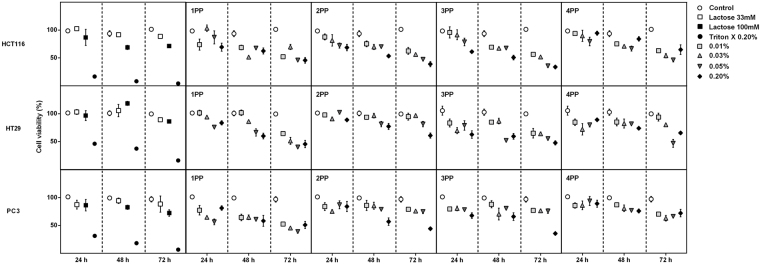
Effects on cell viability of papaya pectin treatment in HCT116, HT29 and PC3. Cells were treated with papaya pectin at different dosages. Papaya pectin decreased HCT116, HT29 and PC3 viability at different levels. The results were expressed in percentage of cell viability in comparison with control (no treatment) of which time. Data were shown as mean ± SD. *P < 0.05 vs control, according to Dunnett’s test. The results were from three independent WSF samples (each one performed in technical triplicate) from the biological duplicate (*n* = 6). PP: papaya pectin (water-soluble fraction).

**Figure 2:**
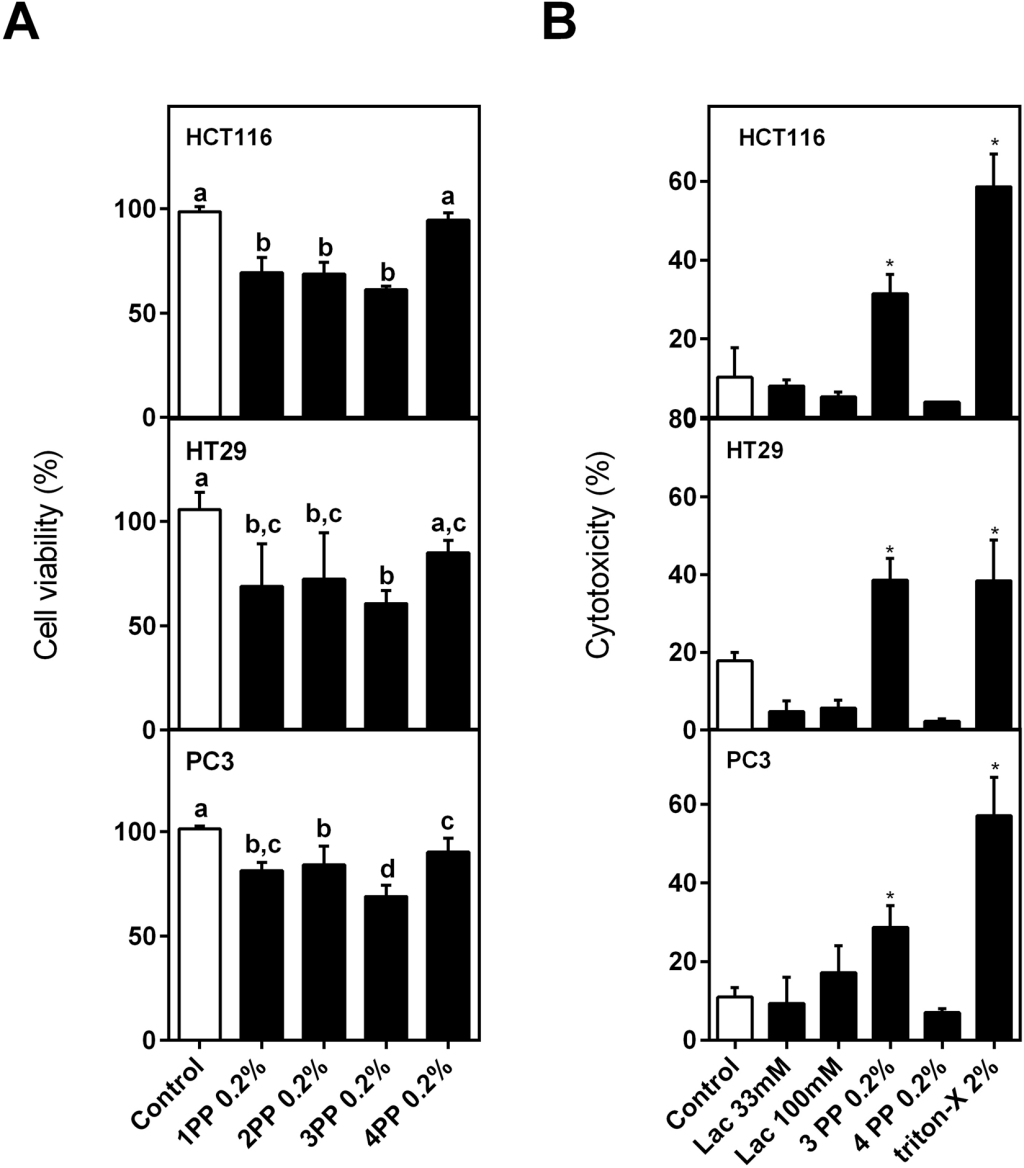
Effects on cell viability and cytotoxicity. (**A**) Effects on cell viability of the higher concentration of papaya pectin t24 h. 3PP strongly reduced cell viability and 3PP and 4PP showed the most distinct results. Data were shown as mean ± SD. Tukey’s test (*P < 0.05) was performed. Different letters represent significant differences between the treatments (as previously explained in Fig. 1). (**B**) Cytotoxicity by LDH assay after 24 h of incubation. 3PP and not 4PP induced cell cytotoxicity. The results were expressed in percentage of cell viability in comparison with control (no treatment) of each time. Results were represented as mean ± SD (as previously explained in Figure 1). *P < 0.05 vs control, according to Dunnett’s test. PP: papaya pectin (water-soluble fraction).

The water-soluble papaya pectin (PP) extracted from different ripening stages of papaya fruit one to four days after harvest (named 1PP, 2PP, 3PP and 4PP, respectively) were screened for viability of HCT116, HT29 and PC3 cancer cells lines (Fig. 1). 3PP and 4PP induced very distinct effects in cells viability after 24 h of treatment (Fig. 2). 3PP (0.20%) induced the highest decrease on cells viability, significantly higher when compared to 4PP (0.20%) **(**Fig. 2A **)**. 3PP was cytotoxic for all cells whereas 4PP was not (Fig. 2B). Because of these striking results with age-dependent changes on the biological effects of pectin, subsequent experiments on cancer cells were done with the 3PP and 4PP at 0.20%.

### 3PP inhibits cancer cells aggregation and migration

**Figure 3:**
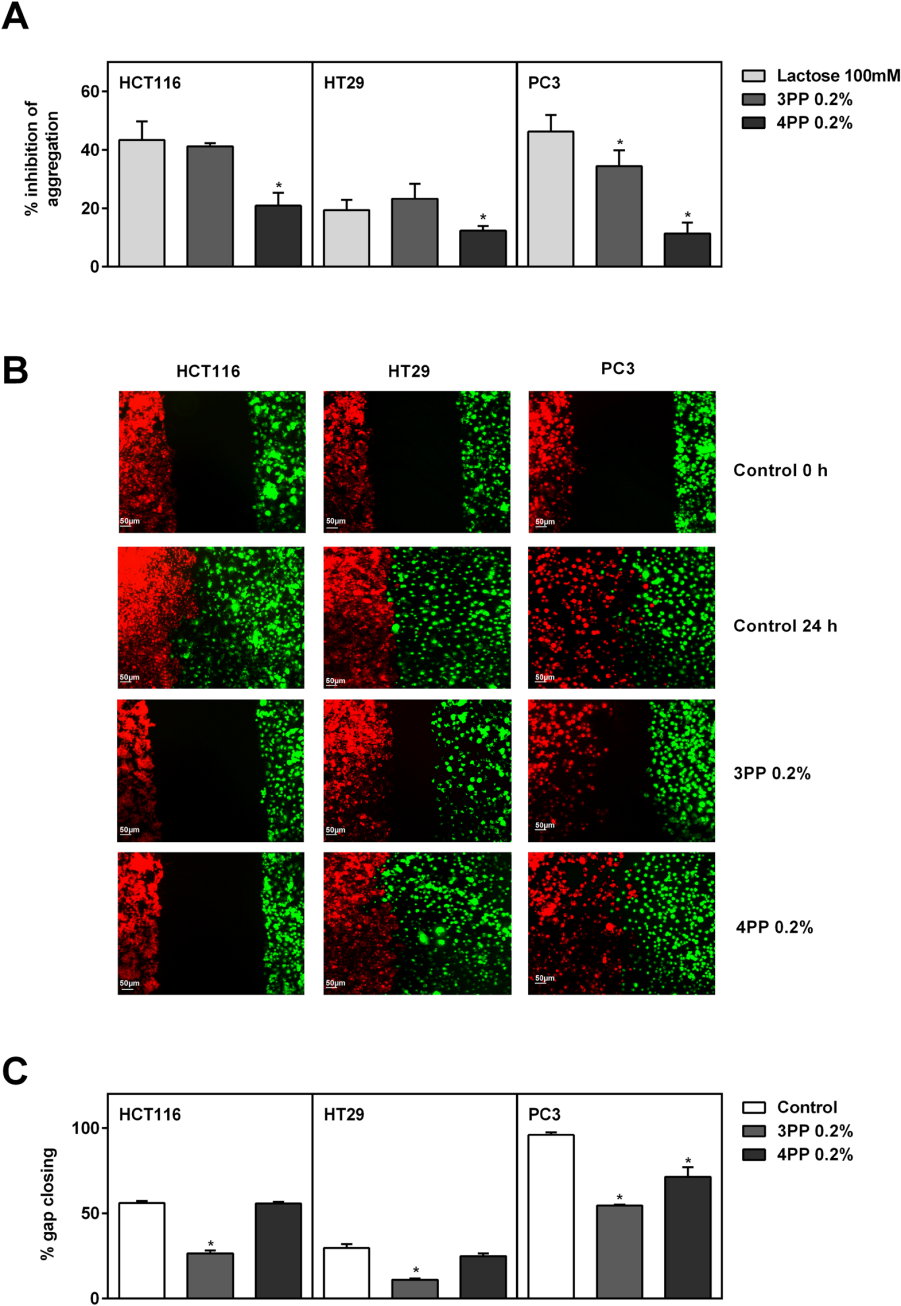
Homotypic aggregation and migration assays (wound healing and endothelial vs cancer cells). (**A**) Inhibition of homotypic cell aggregation using asialofetuin treated with lactose or papaya pectin at 0.2%. 3PP strongly inhibited cancer cells aggregation. The results were expressed in percentage of cells in relation to control (with asialofetuin and no treatment). Data were shown as mean ± SD from two independent WSF samples, each one performed in technical duplicate, from the biological duplicate (*n* = 4). *P < 0.05 vs lactose, according to Dunnett’s test. Images of homotypic aggregation test were in Supplementary Figure S1. (**B**) Endothelial cells (BAMEC) dyed with DiO (green) and cancer cells dyed with DiI (red). 3PP diminish the interaction between cancer cells and BAMEC. Scale bar: 50 µm. Representative image of, at least, two experiments from the biological samples. (**C**) Quatification of gap closing after 24 h. 3PP slowest gap closing compared with control and with 4PP. The results were expressed in percentage of cells that invaded the gap compared with control. Data were shown as mean ± SD (as previously explained in Figure 3A). *P < 0.0001 vs control (without treatment), according to Dunnett’s test. Images of wound healing were in Supplementary Figure S2. PP: papaya pectin (water-soluble fraction).

Beta-D-lactose (in 100 mM concentration) is widely used as a non-cytotoxic cell aggregation inhibitor or apoptosis-inducing in the cancer cells in these experiments (Fig. 2B; Supplementary Figure S3)^33,34^. 3PP and β-D-lactose (100 mM) inhibited the homotypic aggregation for all cell lines with HCT116 and PC3 cells showing the highest inhibition rates (Fig. 3A, Supplementary Figure S1), indicating a possible interaction between 3PP sample with cancer cells. PC3 cells showed lower rates of aggregation after 3PP treatment rather than β-D-lactose and the inhibition of cell aggregation by 3PP treatment was higher than 4PP for all evaluated cell lines. Moreover, cancer cells were assessed for inhibition of migration after papaya pectin treatments. Because growth and spread of cancer cells is dependent on the interaction between cancer cells and endothelial cells,^35,36^ it was tested the interaction of endothelial cells BAMEC (bovine adrenal medullary endothelial cells) with cancer cells, as well as the interaction between cancer cells in the wound-healing experiments (Fig. 3B,C, Supplementary Figure S2). Compared with the control (no treatment), 3PP treatments showed the slowest cell migration for endothelial cells and slower gap closing in wound healing assay after 24 h. 4PP treatment had only slowed cancer cell migration in direction of BAMEC for HCT116 cell treatments and had significantly diminished gap closing for PC3 cells, but with slower rates than for 3PP.

### Papaya pectin affects interaction between cancer cells and extracellular matrix proteins

**Figure 4:**
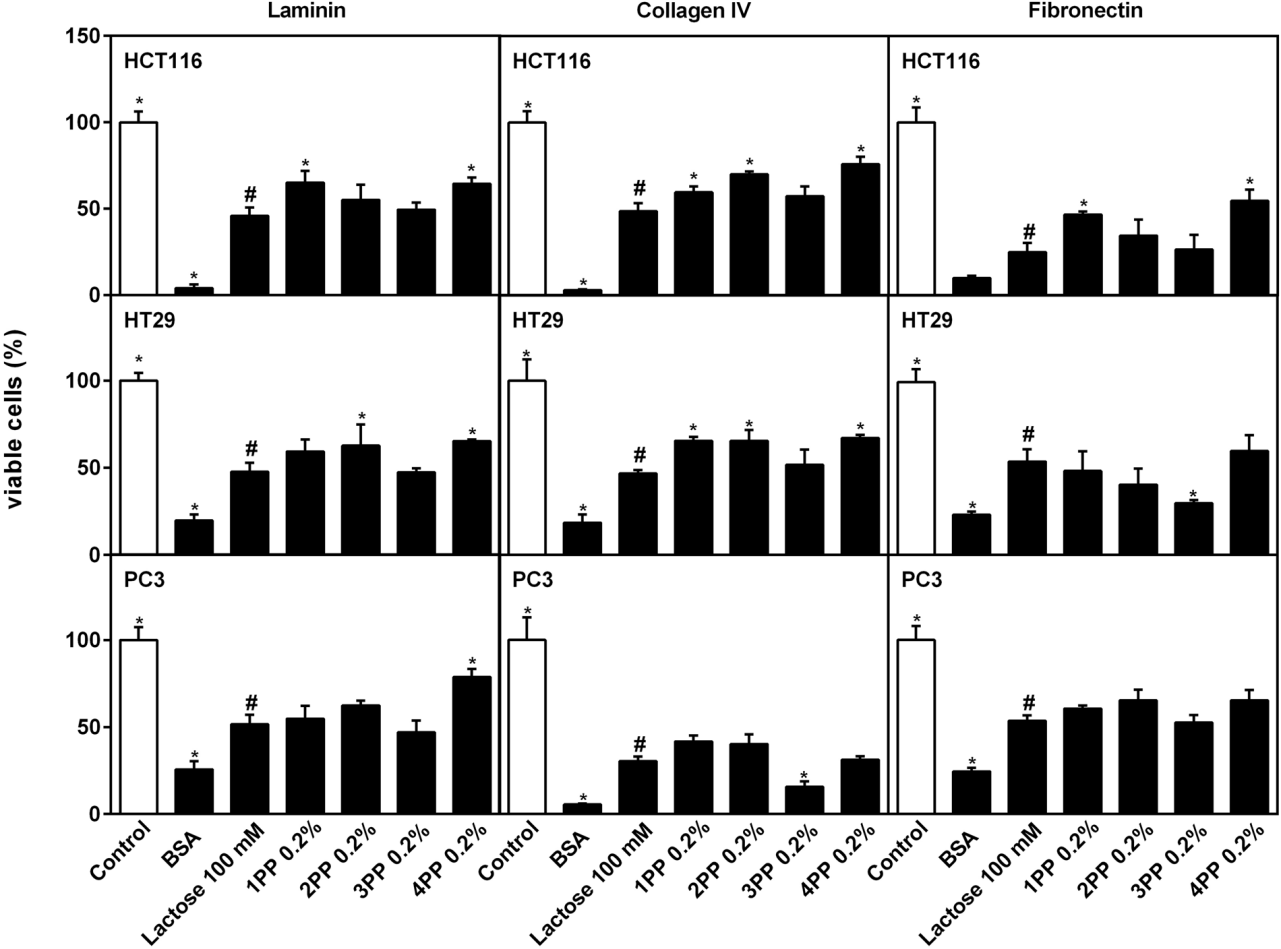
Extracellular matrix proteins (laminin, collagen IV and fibronectin) interactions with cancer cells lines with ou without papaya pectin treatment. Papaya pectin affects interaction between cancer cell and proteins from ECM. The results were expressed in percentage of cells in comparison with control. Data were shown as mean ± SD from two independent WSF samples, each one performed in technical quadruplicate, from the biological duplicate (*n* = 4). All treatments were significant different from control (Dunnett’s test). All samples were compared with lactose (#) by Dunnett’s test and significant differences (P < 0.05) are marked with an asterisk. PP: papaya pectin (water-soluble fraction).

An evaluation of interactions of cancer cell lines with extracellular matrix (ECM) proteins laminin, collagen IV and fibronectin was performed to evaluate if papaya pectin were disturbing cancer cells attachment. In this assay, the numbers of cells that could bind to the ECM proteins pre-coated on cell culture plates and cells viability were assayed. Untreated cells were the control (100% viability/attachment), and cells poured in BSA-coated wells were the negative control of interaction (without proteins of ECM). All treatments lowered cell interactions with ECM proteins significantly from untreated control cells, indicating that papaya pectin affected interactions between cancer cells and ECM proteins (Fig. 4). As lactose is known to interact with galectins to inhibit binding of ECM proteins to cancer cells, data from papaya samples were statistically compared with β-D-lactose treatment. Behaviors of HCT116 cells with 3PP were equal to those treated with lactose treatment for attachment of viable cells to all ECM proteins tested, except for fibronectin in HT29 cells and collagen IV in PC3 cells, where affects were lower than with lactose. 4PP treatments yielded equal or significantly higher attachment than did lactose, indicating they were less effective in inhibiting interactions of cancer cells and ECM proteins. Notably, the lowest attachment values from the interaction between fibronectin and HCT116 and HT29 cells, and between collagen IV and PC3 cells, predict interference of papaya pectins extracted from the third day after harvesting (3PP) on binding of ECM proteins to cancer cells, possibly resulting in cell detachment and cell death during growth.

### ‘3PP’ induces late apoptosis/necroptosis on cancer cells

**Figure 5:**
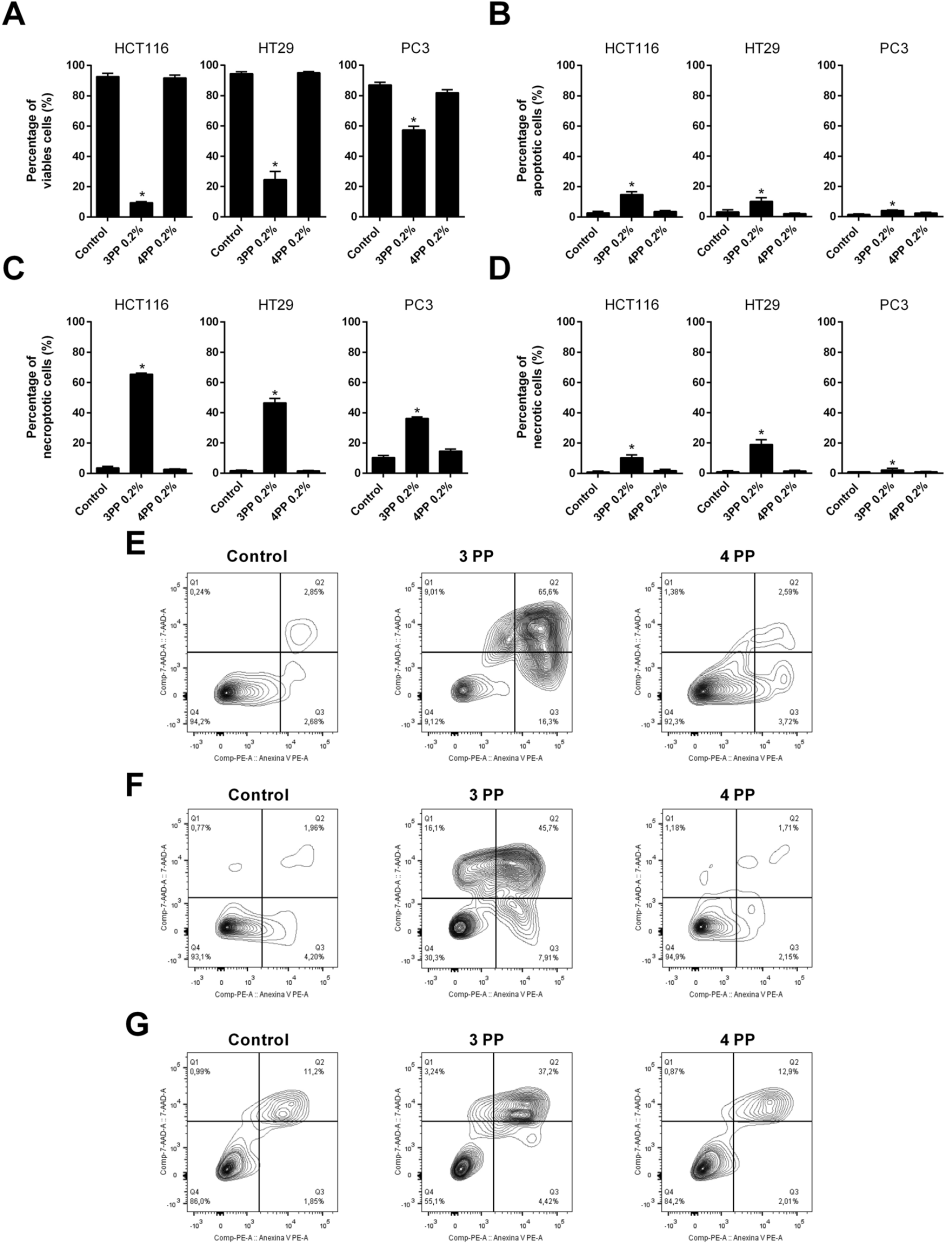
Effects of papaya pectin in HCT116, HT29 and PC3 apoptosis by flow cytometry. Cancer cells had induced late apoptosis/necroptosis with 3PP. Cells were treated with 0.20% of 3 PP and 4 PP pectin for 24 h. (**A**) Percentage of viable cells. (**B**) Percentage of apoptotic cells. (**C**) Percentage of necroptotic cells. (**D**) Percentage of necrotic cells. (**E**) Flow cytometry plots of HCT116. (**F**) Flow cytometry plots of HT29. (**G**) Flow cytometry plots of PC3. The results were expressed in percentage of cells in comparison with control (no treatment). Results were represented as mean ± SD of two independent WSF samples, each one performed in technical triplicate, from the biological duplicate (*n* = 4). *P < 0.05 vs control, according to Dunnett’s test. PP: papaya pectin (water-soluble fraction).

**Figure 6:**
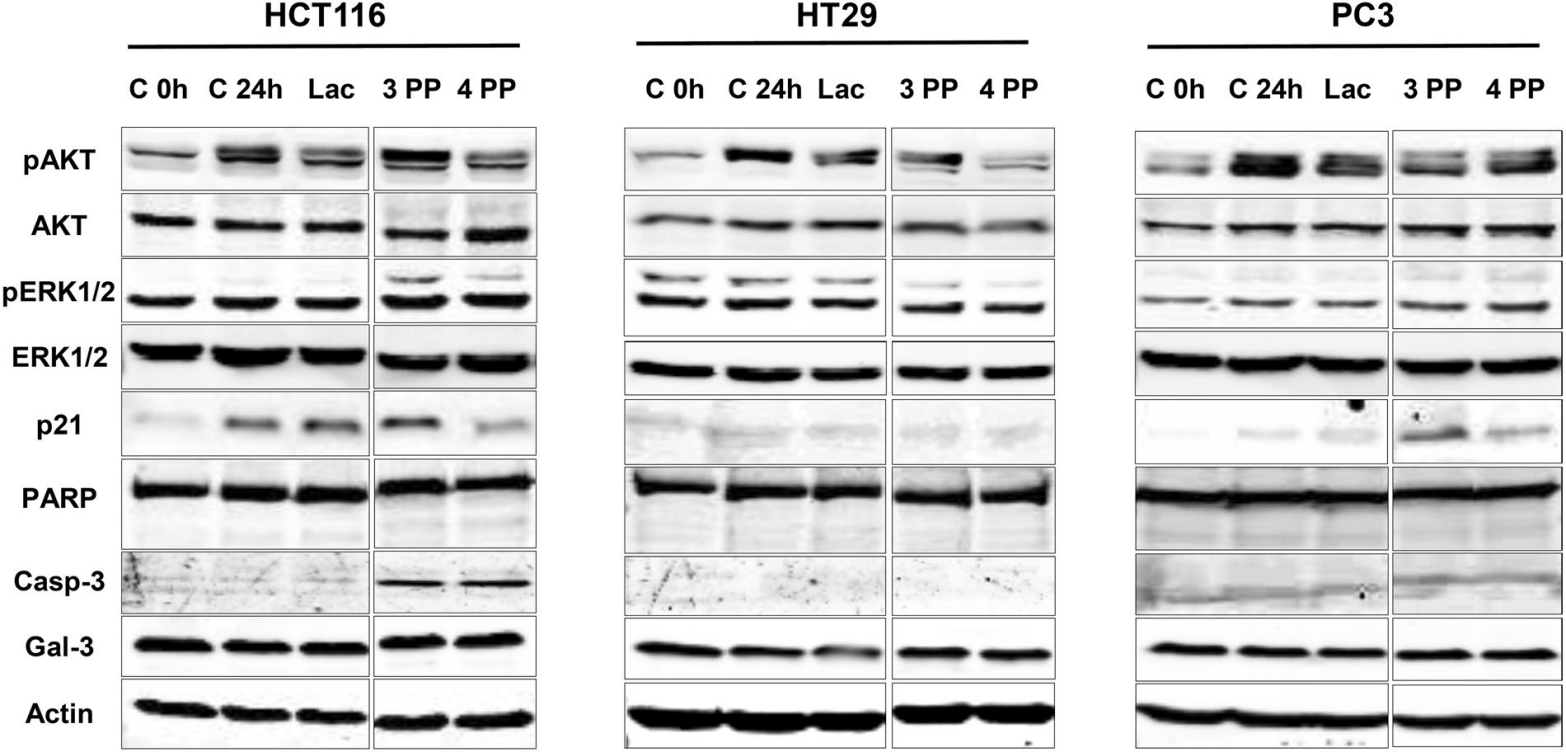
Western blot analysis. Mechanisms of papaya pectin vary among different cell line. Cell lysates were prepared and processed for western blot assay after 24 hours of treatments. After BCA assay, equal amounts of proteins were separated using SDS-PAGE. β-actin was used as the loading control. PP: papaya pectin (water-soluble fraction).

To explore by which mechanisms papaya pectin induced cancer cells death, a flow cytometry analysis was performed in order to verify cell viability and the induction of cell apoptosis, late apoptosis/necroptosis and necrosis through PE Annexin V and/or 7-AAD staining and results were compared to the Western Blotting analysis. 3PP showed higher amounts of late apoptosis/necroptosis events (Fig. 5). In addition, the decrease on cell viability was stronger on HCT116 and HT29 cells. In contrast, 4PP and lactose had no effects when compared to control (Fig. 5, Supplementary Figure S3). Differential protein blotting indicated that the anti-cancer mechanism of papaya pectin varied among different cell lines (Fig. 6). The induced apoptosis after 3PP treatment in HCT116 could be related with caspase 3 pathways activation, despite the fact that 4PP treatment also induced caspase 3 and did not show significant apoptosis. The 3PP treatment increased phosphorylated Akt (pAkt) and phosphorylated Erk1/2 (pErk1/2) protein levels in HCT116 cell line, whereas the opposite effects were observed in HT29 cells. Increased expression of pAkt and cell death for HCT116 cells after 3PP treatment might be related to rapid reactive oxygen species (ROS) accumulation^37^. After 4 h and 24 h of treatment, ROS accumulation was significantly higher in 3PP (Supplementary Figure S4). 3PP, and to a lesser extent 4PP, decreased pAkt and increased p21 expression in PC3 cells. Intracellular quantities of galectin-3 were not affected by treatments. Action of papaya polysaccharides on extracellular area likely do not influence endogenous galectin-3 expression in a short period of time (24 h treatment).

### Papaya pectin characterization

**Figure 7:**
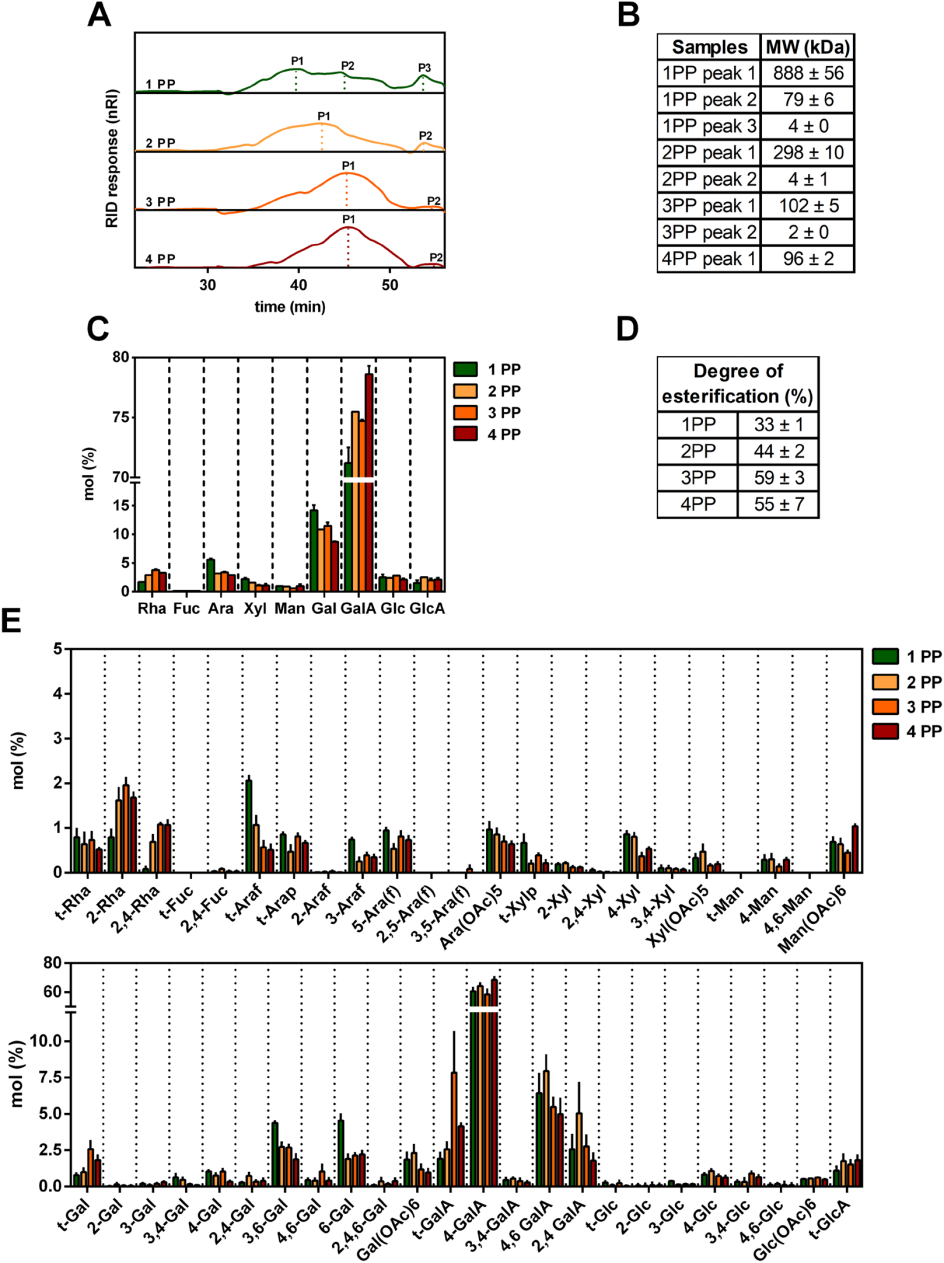
Profile and composition of water-soluble fraction isolated from papaya during 4 days after harvesting. (**A**) HPSEC-RID elution profile. 1PP and 2PP had similar profiles and higher molecular weight compared to 3PP and 4PP. (**B**) Molecular weight was estimated using a standard curve of dextran T-series (5, 25, 50, 80, 150 and 410 kDa; technical triplicate, from the biological duplicate). (**C**) Monosaccharides composition. Papaya pectin is composed mainly by GalA, Gal, Rha and Ara, at different proportions depending on the ripening stage. Results represents mean ± SD (at least seven technical replicates from a pooled WSF triplicate from the biological duplicate; *n* ≥ 7). (**D**) Degree of O-Methyl Esterification. Papaya pectin had esterification increased during ripening. Values were calculated using the calibration curve (R^2^ = 0.9798) and results are expressed in mean ± SD (three technical replicates from a pooled WSF triplicate from the biological duplicate; *n* = 3). (**E**) Water-soluble fraction polysaccharides linkage analysis. Papaya pectin 4-GalA indicates presence of homogalacturonan with reasonable amounts of 2,4-, 4,6-GalA and 2-, 2,4-Rha linkages that are related to type I rhamnogalacturonan. Error bars indicate SDs of the mean (at least seven technical replicates from a pooled WSF triplicate from the biological duplicate). The table of likage results was in Supplementary Table 1. Rhamnose (Rha); fucose (Fuc); arabinose (Ara); xylose (Xyl); mannose (Man); galactose (Gal); Galacturonic acid (GalA); glucose (Glc); glucuronic acid (GlcA); terminal (t); pyranose (p); furanose (f). PP: papaya pectin (water-soluble fraction).

The extraction yields and water-soluble papaya pectic polysaccharides structures were evaluated during the four phases of ripening. Total cell wall yields (TCW) were unchanged throughout ripening. Soluble pectins extracted from different ripening stages increased sequentially from 4.1 ± 1.7% of TCW at phase 1, 12.3 ± 3,3% at phase 2, 25.1 ± 3,7% at phase 3 and 28.7 ± 1.9% at phase 4. Molecular weight distributions analyzed by HPSEC-RID showed that polysaccharides from 1PP and 2PP had similar profiles, but with higher molecular weights, 888 ± 56 kDa and 298 ± 10 kDa, respectively, compared to 102 ± 5 kDa and 96 ± 2 kDa for 3PP and 4PP (Fig. 7A and B). Papaya pectin comprised mostly GalA, Gal, Rha and Ara, demonstrating those fractions were mainly formed by pectin (Fig. 7C). The amounts of GalA and Rha in the solubilized fractions increased throughout ripening, while amounts of Gal and Ara decreased. The degree of esterification has also increased during the course of ripening (Fig. 7D), demonstrating that pectinesterases (PME) are not actively de-methylating pectins after fruit harvesting, corroborating what it has already been proposed elsewhere^25^. This increase in methylation could be explained by the enrichment of pectins in water soluble fraction after being solubilized from insoluble fractions after massive action of polygalacturonases as it had already been stated^25,26^. Linkage analysis (Fig. 7E; Table S1) revealed a high proportion of *4*-GalA indicating higher presence of HG with reasonable amounts of *2*,*4*-, *3*,*4*-, *4*,*6*-GalA and *2*-, *2*,*4*-Rha linkages that are related to the RG-I presence and 3,*4*-GalA indicating the presence of xylogalacturonan^38^. RG-I galactans side-chains were found by the identification of *4*-, *3*,*4*- and *4*,*6*-Gal linkages. Type II arabinogalactans (AGII) appear to be the predominantly ramification of RG-I in papaya pulp pectin structures. AGII structures consist in backbones of *1*,*3*- β-D-and *1*,*6*-β-D-galactan chains with *3*,*6*-Gal branch points and substituted with mostly *t*-Ara*f* at *O*-*6* and *O*-*3* position of the available Gal residues^39^. To estimate the variation ratios of HG and RG-I regions during ripening, data from monosaccharide composition and linkage analyses were summed and compared as GalA:Rha ratio for proportions of HG and RG-I, and Gal:Rha and Ara:Rha ratios for proportions of neutral sugars side chains attached to RG-I (Table 1). The decrease in the ratio of GalA:Rha during ripening indicated an increment of RG-I papaya pectin from ripen fruits. In contrast, Gal:Rha and Ara:Rha decreased during ripening, and this decrease was accompanied by a decrease in proportions of *4*-Gal and *3*,*4*-Gal, indicating loss of galactan and type I arabinogalactan side-chains of RG-I. Persistence of *t*-Ara*f* and 5-Ara*f* during ripening demonstrated selective arabinogalactan/arabinan depolymerization by an arabinofuranosidase. The highest amounts of *2*- and *2*,*4*-Rha in the soluble fractions of 3PP and 4PP, and increased ratios of *2*,*4*-Rha:*2*-Rha during ripening indicated that the RG-I backbone was depolymerized and solubilized to a greater extent than was HG^26^. Ratios of *3*-, *6*-, and *3*,*6*-Gal:*t*-Gal were lowest in 3PP and 4PP, indicating smaller sizes of AGII. The lowest ratio of *4*-, *2*,*4*-, and *3*,*4*-GalA:*t*-GalA in 3PP demonstrated that HG backbone was shortest in 3PP than in 4PP.

**Table 1:** Monosaccharide and linkages ratios of water-soluble fraction isolated from papaya during 4 days after harvesting.

	Ratio of monosaccharides			Ratio of linkage analysis				
	GalA:Rha	Gal:Rha	Ara:Rha	totalGalA: *t*-GalA	*4*-, *2*,*4*,*3*,*4*-GalA: *t-*GalA	*3-*, *3*,*4-*, *4-*, *4*,*6-*, *3*,*6-*, *6-*Gal: *t*-Gal	*2*,*4*-Rha: *2*-Rha	*3-*, *6-*, *and 3*,*6-Gal: t-Ga*
1 PP	41.9	8.4	3.3	36.0	33.1	14.0	0.1	11.5
2 PP	26.0	3.8	1.1	30.0	27.2	6.0	0.4	4.8
3 PP	19.7	3.0	0.9	9.0	7.8	3.0	0.6	1.9
4 PP	23.8	2.6	0.9	18.0	17.0	3.0	0.6	2.4

PP: papaya pectin (water-soluble fraction); GalA: galacturonic acid; Rha: rhamnose; Gal: galactose; Ara: arabinose.

**Figure 8:**
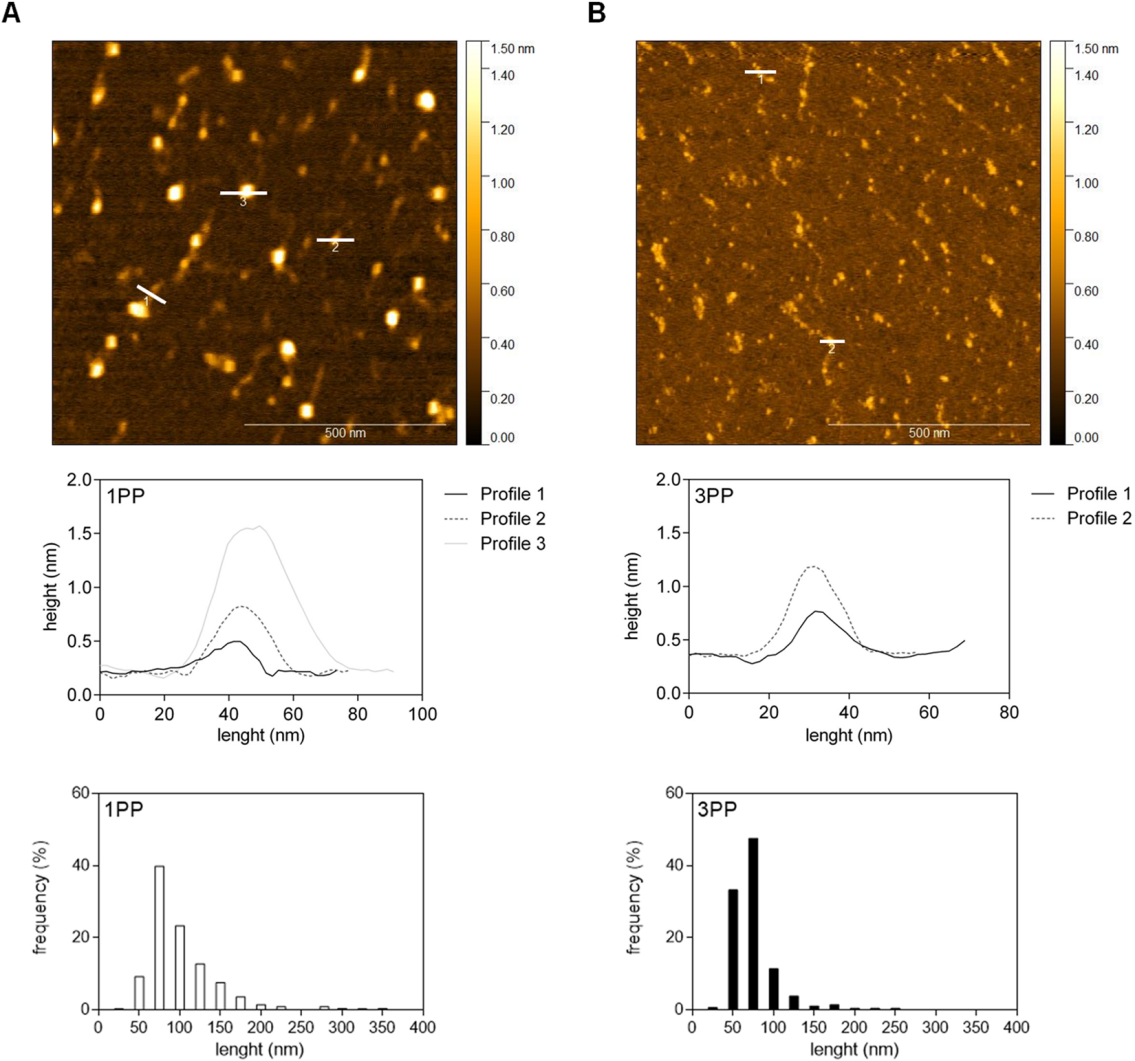
Representative topographical AFM images of papaya water-soluble pectins. (**A**) 1PP sample topography, height profile and length frequency. 1PP had linear chains and micellar aggregates. (**B**) 3PP sample topography, height profile and length frequency. 3PP had both chains length and micellar aggregates decrease. PP: papaya pectin (water-soluble fraction). Representative image of, at least, two experiments from the biological samples.

Because size distributions and branching patterns varied widely among 1PP and 3PP, we investigated their ultrastructural features through atomic force microscopy in order to compared unripe and ripe fruits with or without biological activity. Side chains were not easily visualized even at higher magnification (1 µm × 1 µm), probably because of the relatively high predominance of HG on papaya pectin (Fig. 8). 1PP was characterized by linear chains and micellar aggregates (Fig. 8A), and 3PP showed a decrease in chain length and micellar aggregates (Fig. 8B). The height profile was similar in both samples, but the higher weight of profile 3 in 1PP sample indicates larger micellar aggregates. Length histogram clearly shows higher frequency of smaller lengths around 50 nm in 3PP.

## Discussion

The softening of papaya fruit is a complex process that occurs during fruit ripening, being coordinated by the action of several cell wall degrading enzymes^25^. In a previous study, we identified a mobilization of high molecular weight pectin from less soluble to more soluble fractions during the papaya ripening because of pectin modification by endogenous pectinolytic enzymes^26^. Several studies have attributed anti-cancer activities to pectins which have been thermally, chemically or enzymatically modified^6,14,16,40–43^. The pectin structure described as responsible for anti-cancer activities are the galactan and arabinan side-chains together with an RG-I/HG backbone^6^. In this regard, papaya pectin previously isolated from different ripening stages seems to have a promising composition concerning the presence of galactans, arabinans, RG-I and HG structures^24,26^. To explore the biological activity of papaya pectin naturally modified by ripening phenomenon, three cancer cell lines were treated with water-soluble papaya pectin extracted from fruits in distinct ripening stages. Initially, papaya pectins were screened for their biological activity on cancer cells. Following this, the mechanisms by which papaya pectins affected the behaviors of cancer cells were explored. Finally, papaya pectins were identified and chemically characterized to pinpoint possible structure-activity relationships.

In a previous work, the key enzymes responsible for physiological degradation of papaya pectins in fruit cell wall were endo-polygalacturonases and exo-galactosidases^25^. Besides, endo-polygalacturonases overexpression during papaya ripening solubilized pectin from the less soluble fractions yielded incremental increases of GalA during ripening^26^. The action of endo-polygalacturonases and exo-galactosidases could explain why *t*-GalA and *t*-Gal linkages increased during ripening. Endo-polygalacturonases would hydrolyze HG in smaller chains, increasing *t*-GalA, while exo-galactosidases could cleave terminal sites of AGII or RG-I thus increasing *t*-Gal and decreasing Gal. Ara also decreased during ripening, while *2*- and *2*,*4*-Rha and the ratio *2*,*4*-Rha:*2*-Rha increased, indicating the presence of smaller chains of more highly branched RG-I. This is the first academic report of AGII structures in papaya pulp. Decreased *4*-Gal and *3*,*4*-Gal indicate less branching with *4*-galactan and type I arabinogalactan side-chains. The increased *2*- and *2*,*4*-Rha and ratio of *2*,*4*-Rha:*2*-Rha during ripening has two possible explanations. Firstly, RG-I depolymerization at the unbranched *2*-Rha linkages through the action of rhamnogalacturonan hydrolases or rhamnogalacturonan lyases (RGases) would shorten chains and increase the ratio of branched to unbranched Rha, but these genes/enzymes have never been identified in papayas. Secondly, as previously reported by our group^26^, enhanced of solubility of HG and RG polysaccharides during ripening occurs. In addition, the significant increase of GalA may have proportionally reduced the Gal and Ara amounts. Besides, the higher expression of an L-arabinofuranosidase during the early days before harvesting^25^ could be responsible for *t*-Ara*f* decreases.

Samples from 1PP had the highest Gal and Ara amounts and the highest proportion of linkages related to AGII. 1PP had also the highest total GalA:*t*-GalA ratios, indicating larger chains of HG and RG-I. The relatively high molecular weight with the lowest yields and difficulty of solubilization of 1PP and 2PP is likely to have interfered with these pectins ability to interact with cancer cells, thus decreasing their biological activity. On the other hand, as described above, the decreased values of total GalA:*t*-GalA ratios in 3PP samples indicated smaller chains with reduced side-chains, thus increasing possible interactions with cancer cells.

Unexpectedly, 4PP had the least anti-cancer activity among all papaya pectins. The reduced anti-cancer activity of 4PP seems to be related to an increment of the total GalA:*t*-GalA ratio as well as the increase of total GalA, indicating higher amounts of longest HG chains when compared to 3PP. The massive endo-polygalacturonase action during fruit ripening can result in the addition of HG in 4PP as endo-polygalacturonases hydrolyze pectins from the less soluble fraction (the ones chelated with calcium with fewer neutral sugar ramifications) to the water-soluble fraction, as previously shown by our group^26^. A qualitative analysis for oligosaccharides was done in order to visualize this massive endoPG activity and the possibility of being some specific oligo that conferred the anti-cancer activity to 3PP. Figure S6 shows 4PP presented a broader detection of oligosaccharides than other pectin fractions with slightly differences, though. This could endorse the generation of oligosaccharides throughout ripening but this was not responsible for the *in vitro* biological activity observed. Contents of starch, proteins and phenolic compounds in the water-soluble fractions of these papaya pectin fractions were insignificant.

Ultrastructural analysis by AFM confirms that 3PP had smaller backbones when compared to 1PP. The micellar aggregates observed mainly in 1PP are characteristic of polymers complexes held together by intermolecular interactions showing pectin heterogeneity and complexity^44^. In a study with strawberry, pectins treated with endo-polygalacturonases showed a decrease in micellar aggregates length, distribution and branching patterns^45^. Thus, the increase of endo-polygalacturonases action during papaya ripening could be responsible for decreasing the pectin length and the micellar aggregates in 3PP. The branched fractions could not be visualized by AFM, because papaya pectins were mainly composed by linear GalA (HG) and the length of neutral sugars branch were not long enough to be easily visualized^46^.

The majority of colorectal cancer cells (53%) show mutation on KRAS, being characterized by the activation of both mitogen activated protein kinase (MAPK) and phosphoinositide 3-kinase (PI3K) signaling pathways^28^. HCT116 had KRAS mutation and possess high metastatic potential compared with HT29^30,31^. PC3 cells had p53 mutation that are more common in prostate cancers of higher tumor stage and metastases^47^. Therefore, cell treatments in the present work could simulate a local effect of papaya pectin ingestion (HCT116 and HT29) or a possible systemic effect (PC3), with results being cell-line dependent.

Although 1PP, 2PP and 4PP significantly affected cell proliferation, samples from 3PP have shown the most striking results. Because 3PP and 4PP treatments (0.20%) showed the highest inhibition in the MTT assay, those samples were chosen for further assays. Although size distribution patterns were similar for 3PP and 4PP, deeper structural analysis revealed that 3PP had an addition of smaller HG and higher ramifications proportions. 4PP showed an increase of a larger HG backbone that could explain the weaker results observed in viability assays. Polygalacturonic acid (PGA), an unbranched HG backbone, had no effect on the viability of colon cancer cells^7^.

Homotypic aggregation was tested with asialofetuin, a glycoprotein with several branched oligosaccharide side chains with terminal non-reducing galactosyl residues^48^. Asialofetuin binds to the lectins galectin-1 and galectin-3, which are located on the surface of cancer cells, and induces homotypic aggregation by serving as a cross-linking bridge between adjacent cells^12^. All cell lines studied here express galectin-3, which induce cell aggregation by interaction with cancer associated MUC1 (large and heavily glycosylated transmembrane mucin protein)^49^. The homotypic aggregation is associated not only with the tumor formation and anoikis resistance, but also with cancer cells heterotypic adhesion to endothelium stimulating metastasis^50^. β-D-Lactose and 3PP showed the highest inhibition of aggregation, which could be related with galectin interaction. Effects of papaya pectin on cell aggregation were more easily visualized with HCT116 and PC3 cells than with HT29 cells. As mentioned previously, HCT116 and PC3 have higher metastatic potential that are related with superior ability to form homotypic aggregates when compared with the less aggressive HT29 cells^51^.

Compared with 3PP, lactose (100 mM) showed less activity in decreasing viability of cancer cells. Because lactose is a classical galectin-3 inhibitor, other targets of papaya pectin exist in addition to galectin-3. Thus, other potential mechanisms of papaya pectin on cancer cells were also screened. 3PP decreased cancer cell migration (endothelial cells vs cancer cell and wound healing assay) without significantly affecting viability of BAMEC endothelial cells. Curiously, 4PP treatment reduced migration of HCT116 and endothelial cells (BAMEC), but HCT116 cell migrated normally in wound healing experiments. The differences in cell migration could be related to the microenvironment that likely plays a crucial role in tumor growth and metastasis^35,36^. Further study will be necessary to evaluate the effects of papaya pectin on the complex interaction between cancer cells and endothelial cells.

The interaction between cells and the extracellular matrix proteins (ECM) is crucial for cell adhesion and migration. We investigated the cell interaction with three glycosylated proteins that comprise the ECM, laminin, collagen IV and fibronectin. In HCT116 cells, fibronectin seems to plays a major role in cell spreading and migration rather than does laminin and collagen^52^. That HCT116 cells did not adhere to culture plates pre-coated with fibronectin after lactose and 3PP treatments supports this conclusion. This could explain why cells treated with 3PP exhibited decreased cell migration, indicating a 3PP-mediated disruption in the interaction between fibronectin-linked glycans and integrins. In addition, the effect of lactose on HCT116 cells in plates pre-coated with fibronectin could indicate a disruption of fibronectin interaction with galectin-3 that could bias fibronectin function on cell migration. Galectin-3 has been shown to modulate fibronectin tumor cell motility^53^. However, as mentioned above, galectin-3 inhibition might not be the main cause of reduced cell migration after treatment with papaya pectins.

Pectin from the third day after harvesting (3PP) was the most effective treatment to cause cell death, increased expression of pAkt and pErk in HCT116 cells. Higher pAkt and pErk expression is usually related with cell migration and survival^54–56^. PI3K/Akt pathway is likely to be a key mediator of fibronectin-integrin effects on cell proliferation^54^, whereas cellular dispersal and motility are known to be mainly regulated by PI3K/Akt and Erk–MAPK signaling pathways^55,56^. On the other hand, pAkt expression is associated with ROS accumulation^37^, and necroptosis can be linked to the metabolic stress caused by partial cell detachment^57^, a probable cause that could explain the 3PP-induced cell necroptosis verified in HCT116 cells. The up-regulation of the Erk pathway could also induce cell necrosis by necroptosis or autophagy responses as previously observed in lung cancer cells^58^. Furthermore, HCT116 *KRAS* mutation has been related with inhibition of caspase-3/7 and promotion of cell viability^59^. 3PP and 4PP, but not lactose, had up-regulated cleaved caspase 3 on HCT116 cells, suggesting the induction of apoptosis. Moreover, activation of p21 may have stimulated apoptosis through both p53-dependent and p53-independent mechanisms as it has already been detected under certain cellular stresses^60^. However, because 3PP led to fewer numbers of apoptotic cells than necroptotic cells, the mechanism of why cells have rapidly passed from apoptosis to necroptosis is still elusive but is clearly dependent on papaya pectin structure.

Results from HT29 cells treatment could be explained by other mechanisms. Integrin consists of two subunits (α and β) of proteins tightly associated with each other^61^. The α5β1 integrin is the fibronectin receptor and the interference in the association between subunits decreases protein binding impairing fibronectin-mediated cell adhesion^62^. 3PP reduced HT29 attachment in plates coated with fibronectin, which is highly expressed in HT29 cells and is associated with cancer cell metastasis^63^. In addition, 4PP, and to a lesser extent 3PP, reduced pAkt expression in HT29 cells. Because 3PP had higher inhibitory activity on cell migration than 4PP, pathways other than p-AKT signaling could be involved in reducing HT29 migration and cell death.

3PP treatment reduced PC3 attachment in plates coated with all three ECM proteins with the stronger results on collagen IV interaction. PC3 cell lines express high levels of integrin subunits associated with collagen binding and lower levels of integrin subunits associated with fibronectin binding. This is the main cause for the enhancement of metastasis guided by cell migration^64^. Because 3PP treatment decreased PC3 cell attachment to collagen IV, papaya pectin might disrupt integrin binding to collagen IV. Moreover, 3PP down-regulated pAkt and up-regulated p21 on PC3 cells. For prostate cancer cells, pAkt expression has a pivotal role in cell migration dependent of epidermal growth factor receptor (EGFR) by activating epithelial–mesenchymal transition^65^. Thus, lower levels of pAkt could be associated with reduced PC3 cell migration and increased cell death. Further, p21 up-regulation in PC3 and in HCT116 cells could be responsible for induction of cell death as it has been observed to glioma and ovarian cancer cells overexpressing p21^66,67^ with a cell-specific sensitivity to oxidative stress, leading to cell death^68^.

The variation of biological activities of papaya pectin obtained from different fruit ripening stages is interesting, as the unmodified citrus pectin and unmodified sugar beet pectin have weak activity on decreased viability of cancer cells^6,12^. Decreasing the average molecular weight by high temperature, alteration in pH, or pectinolytic enzymes improve the anti-cancer activities. Low-molecular-weight modified citrus pectins (1% w/v) are associated with the inhibition of gastrointestinal cancer cells growth and metastasis^11^. Sugar beet pectin modified by alkali treatment (0.05% or 0.1%; for 72 h) induced apoptosis in HT29 colon cancer cells via an interaction with the neutral sugar side-chains from the RG-I^6^. A similar pectin structure from ginseng described as a RG-I-rich polysaccharide with (1,4)-β-D-galactan side-chains showed high affinity to galectin-3 revealed by the hemagglutination assay^19^. Although some researchers proposed that pectin anti-cancer activities can be explained by the binding to the carbohydrate recognition domain of extracellular galectin-3 binding^12,69^, the precise mechanisms remain unclear. Morris *et al*.^70^ suggested that pectin with charged GalA residues attached to the RG-I backbone could be responsible for a non-specific binding to galectin-3 through non-specific charge–charge interactions^70^. However, LNCaP prostate cancer cells (a non-expressing galectin-3 cell line) treatment with pectins showed some apoptotic effects which were due to mechanisms not mediated by galectin-3 inhibition^13^. Another study suggests that polysaccharides with content of RG-I and HG variation could inhibit colon cancer cells proliferation by decreasing ICAM1 expression, a protein responsible for cell–cell interaction and cell–ECM interaction independent of galectin-3 expression^7^. In our experiments, none of the papaya pectins inhibited the hemagglutination mediated by recombinant human galectin-3 (even at high concentrations), demonstrating that the biological activity observed herein for papaya pectins is independent of galectin-3 inhibition.

In summary, we show here that papaya pectin compositions and structure are affected by the coordinated action of several pectinolytic enzymes during fruit ripening. Those changes in pectin structures influenced the possible structure-activity relationships when cancer cell lines were treated with naturally modified papaya pectin. We observed that 3PP has increased the anti-cancer activity when compared to pectin isolated from other ripening stages. These differences could be related, at least in part, because 3PP had smaller HG chains, smaller RG-I side-groups, and AGII associated with RG-I. It was the first time that these pectin structures were isolated and identified in papaya fruit. The inhibitory effects of the smaller neutral chains of RG-I identified in ripe papaya pulp on the interaction of ECM proteins laminin, collagen IV and fibronectin with cancer cells that might be the causal cancer cell death. As different mechanisms of cell death and migration are affected in cancer cells, additional studies regarding the signaling pathways of migration and cell death should be performed. Differences in papaya pectin structures and the different cell types used in these experiments would exhibit the distinct biological effects on cancer cells treatments. The changes in the structure of the papaya pectin driven by natural ripening provide promising clues regarding the structures of bioactive fruit compounds, especially bioactive polysaccharides that are found in fleshy fruit.

## Materials and Methods

### Antibodies, Chemicals, and Reagents

Heat-inactivated fetal bovine serum (FBS), trypsin / EDTA and Dulbecco’s modified Eagle’s medium (DMEM) containing penicillin (100 UI/mL) and streptomycin (100 μg/mL) were from Gibco (Grand Island, NY) or Cultilab (Campinas, SP). Unless stated otherwise, other reagents and chemicals used were from Sigma-Aldrich (St. Louis, MO). P-Akt (sc-7985-R), p-Erk (sc-7383) and Erk 1/2 (sc-135900) were purchased from Santa Cruz Biotechnology (Santa Cruz, CA). Akt (#9272), p21 (2946S), PARP (#9542), cleaved caspase-3 (#9661) antibodies were purchased from Cell Signaling Technology (Beverly, MA). Monoclonal rat anti-Gal-3 antibody was isolated from the supernatant of hybridoma (catalog number: TIB- 166, American Type Culture Collection; Manassas, VA). Mouse anti-β-actin was purchased from Sigma-Aldrich.

### Plant material

Papaya fruits (*Carica papaya* L. cv. ‘Golden’) were acquired from a producer in Linhares City, Espírito Santo, Brazil (19°21′33.9″S 40°08′15.6″W). The fruits were harvested at color break to one-fourth yellow and stored at ambient temperature during ripening. Respiration, ethylene production and pulp firmness were measured daily from, at least, six fruits^25^. Fruit pulps were cut in small pieces, frozen in liquid nitrogen, pooled and stored (−80 °C) at each day for further analysis^25^. For the following analysis, it was used different phases of ripening, separated by one to four days after harvesting from two independent ripening curves (biological duplicate) as previously stated^25^.

### Extraction of water-soluble fraction

The frozen papaya pulp was grounded to fine powder in N_2_ and extracted three times (or until samples were colorless) with chloroform:methanol (1:1; v/v) for enzyme inactivation and protein/pigment removal. Residues were washed with three volumes of 80% boiling ethanol for monosaccharide removal and were also washed with three volumes of acetone for drying purposes. Finally, residues from triplicate extractions were dried and weighed, resulting in a total cell wall fraction (TCW). Extraction yields were achieved using the total pulp values used in experiments. Then, each TCW sample was extracted three times with deionized water under constant magnetic stirring for 20 min at 25 °C and centrifuged (10,000 × *g*, 20 min, 25 °C). The supernatant (water-soluble fraction-WSF) was lyophilized and weighed for extraction yields calculation related to TCW quantity. Qualitative analysis for starch, proteins and phenolic compounds were performed in order to test the purity of WSF fractions.

### Cell culture and treatments

Colon cancer cells line HCT116 and HT29 and prostate cancer cell line PC3 were purchased from American Type Culture Collection guidelines (ATCC) or Rio de Janeiro Cell Bank (BCRJ, Rio de Janeiro, RJ). Cell lines were cultured in Dulbecco’s modified Eagle’s medium (DMEM) containing penicillin (100 µL/mL) and streptomycin (100 μg/mL) with 10% fetal bovine serum and cells were maintained at 37 °C in a humidified atmosphere of 95% air and 5% CO_2_. The ATCC for the maintenance of cells were followed. Cells were dissociated from growth dishes by using trypsin/EDTA when reached a confluence of 70–90%. For treatment, 0.22 µm filtered WSF (0, 0.013, 0.025, 0.05 or 0.2%), lactose (33 or 100 mM) or Triton-X (0.2%) was added to the medium at those final concentrations.

### MTT assay

Cells were plated on a 96-well cell culture plates at a density of 1 × 10^4^ cells/well (200 μL) overnight. For MTT assay cells were incubated with the culture medium (control) or papaya WSF (0.013, 1.025, 0.05 or 0.2%) for 24, 48 and 72 h. After incubation, MTT (0.5 mg/mL) was added for 3 h. Supernatants were removed and the formazan crystals were solubilized with DMSO. Absorbance at 490 nm was measured in a microplate reader (Bio-Rad, Hercules, CA). Cells viability at each incubation time was expressed in relation to the untreated cells (control).

### LDH assay

The lactate dehydrogenase (LDH) was evaluated using the Cytotoxicity Detection Kit (Roche Diagnostics, Mannheim, Germany) following the manufacturer’s instructions. Briefly, cells were plated overnight as described for viability assays and incubated with the culture medium (control) or polysaccharides. After incubation, supernatants (100 µL) were transferred to 96-well cell culture plates, mixed with the Substrate solution and incubated at 25 °C for 30 min protected from light. Finally, the stop solution was added and the absorbance at 490 nm was measured in a microplate reader (Bio-Rad). The cytotoxicity (%) was expressed as the amount of LDH released by cells in relation to cells treated with a Lysis solution. Only samples from 3PP and 4PP were analyzed because of the prominent difference in the initial cell proliferation assays.

### Homotypic aggregation assay

The assay was performed as described by Nangia-Makker, Balan and Raz^48^. Cells were detached from monolayer with 0.02% EDTA in calcium-Magnesium free PBS (CMF-PBS) and suspended at 1 × 10^6^ cells per mL in CMF-PBS with or without 20 g/mL asialofetuin. Samples were placed in 0.5 mL aliquots into siliconized glass tubes and agitated at 80 *g* for 60 min at 37 °C. The aggregation was terminated by fixing the cells with 1% formaldehyde in CMF-PBS. Single cells were counted in a TC10™ Automated Cell Counter (Bio-Rad) and the percentage of aggregation inhibition was calculated as suggested by the above-referred article.

### Migration assay

Migration assay was performed as described by Nangia-Makker, Balan and Raz^48^. Briefly, bovine adrenal medullary endothelial cells (BAMEC) or HCT116, HT29 and PC3 cells (2.4 × 10^4^) were seeded in each chamber of the cell culture insert (Ibidi GmbH). BAMEC were maintained in Earle’s Minimal Essential Medium (EMEM - Invitrogen, Carlsbad, CA) containing 10% heat-inactivated FBS, 2 mM glutamine and antibiotics. Cells were prelabeled with DiO (green) or DiI (red) (Invitrogen, Carlsbad, CA). After 12 h, the cell culture insert was removed, cells were washed with PBS and EMEM medium without FBS and with the respective components was loaded and the cell migration of the co-cultures towards each other was observed after 24 h under fluorescent microscope and compared to a previous 0 h observation to analyze migration. Photos were taken using a Zeiss Confocal Laser Microscope LSM 510 META NLO (The Wayne State University Microscopy and Imaging Core Facility). Only samples from 3PP and 4PP were analyzed because of the prominent difference in the initial cell proliferation assays.

### Wound healing assay

Wound healing assay was performed as described by Moreno-Bueno *et al*. (2009). Cancer cells were plated (2 × 10^5^) in 35-mm cell culture dishes in 1.5 mL growth medium without FBS. Cells were incubated in a humidified incubator at 37 °C, 5% CO_2_ until reached 85% confluence. A wound was made by scratching the monolayer culture using a sterile 200 µL micropipette tip. Afterwards, cells were washed with PBS to remove floating cells and 1.5 mL of culture medium was added with or without pectin treatments. Only samples from 3PP and 4PP were analyzed because of the prominent difference in the initial cell proliferation assays.

### Extracellular matrix proteins

Extracellular matrix proteins interactions were performed according to Nangia-Makker, Balan and Raz^48^. Firstly, a 96-well microtiter cell culture plates were coated with serially diluted (0 to 10 μg) EHS laminin, collagen type IV, or fibronectin. Plates were incubated for 1 h at 37 °C to dry the ECM protein. The non-specific sites were blocked in the wells by incubating with sterile 1% BSA in PBS for 1 h at 37 °C. Wells were washed with sterile PBS three times to remove extra proteins. Cells were detached from the plate using 0.02% EDTA. Viable cells were counted using trypan blue and seeded at 4 × 10^4^ cells per well. Cells were allowed to adhere to the plates for 16 h. Non-adherent cells were washed off with medium three times. To count the number of cells attached to the ECM proteins was added 200 mL of the medium and a 1:10 dilution of Alamar blue was added. The live cells created a reducing environment, which changed the color of dye from blue to pink. Cells were incubated for 3 h at room temperature and read absorbance at 570 nm and with a fluorescence excitation at 570 nm, fluorescence emission was read at 585 nm. After achieving the best ECM quantity for each well (1 µg for laminin, 0.5 µg for collagen IV and 2.5 µg for fibronectin), the same experiment described above was done but using the different cells treatment (100 mM lactose and 0.2% papaya pectin – 24 h) with no coated proteins as positive control and 0.1% BSA-coated wells as negative control.

### Apoptosis assay

Cells were plated on a 24-well cell culture plates at a density of 2 × 10^5^ cells/well (1 mL) overnight. Then, cells were incubated with the culture medium (control), WSF (3PP and 4PP; 0.2%) or lactose (100 mM) for 24 h. Apoptosis was evaluated using PE Annexin V Apoptosis Detection Kit I (BD Biosciences, San Diego, CA) according to the manufacturer’s instructions. Briefly, cells were washed twice with cold PBS with 2% BSA and then ressuspended in 1x Binding Buffer at a concentration of 1 × 10^6^ cells / mL. A solution of 1 × 10^5^ cells (100 µL) was transferred to a 5 mL culture tube. FITC Annexin V (5 µL) and 7AAD (5 µL) were added in the tube, gently vortex and incubated for 15 min at RT (25 °C) in the dark. Finally, 400 µl of 1x Binding Buffer were added to each tube and analyzed by flow cytometry within 1 h using a FACSVerse flow cytometer (BD Biosciences, San Diego, CA). Controls of unstained cells and staining only with FITC Annexin V or 7AAD were used. Data analysis was performed with FlowJo software (Tree Star, Ashland, OH). Only samples from 3PP and 4PP were analyzed because of the prominent difference in the initial cell proliferation assays.

### Western blotting assay

Cells were plated on a 6-well cell culture plates at a density of 5 × 10^5^ cells/well (2 mL) overnight. Then, medium was changed and new medium with lactose (100 mM), 3PP (0.2%), 4PP (0.2%) or without any source of carbohydrate were added and left at incubation for 24 h. The medium was discarded and cells were washed with PBS twice (0 and 24 h of treatment). Cells were lysed in RIPA buffer (50 mM Tris-HCl pH 7.4, 1% NP-40, 0.5% Na-deoxycholate, 0.1% SDS, 150 mM NaCl, 2 mM EDTA, 50 mM NaF and 0.2 mM Na_3_VO_4_) containing protease and phosphate inhibitors (Roche Applied Science, Nutley, NJ). BCA protein assay (Pierce Biotechnology, Rockford, IL) was performed to determine equal amounts of proteins for 8% or 10% SDS-polyacrylamide gel electrophoresis (PAGE) and transferred to polyvinylidene fluoride membranes (Millipore, Bedford, MA). Membranes were blocked in 0.1% casein/Tris buffered saline (TBS) for 1 h, incubated with appropriate primary antibodies for overnight at 4 °C, and after, incubated with secondary antibodies conjugated with IRDye 800 (Rockland Immunochemicals, Gilbertsville, PA) or Alexa Fluor 680 (Invitrogen, Carlsbad, CA) for 1 h at room temperature. Membranes were washed three times with TBS including 0.1% Tween20 at 5 min intervals, and were visualized using an Odyssey Infrared Imaging System. Each experiment was repeated at least, twice. Only samples from 3PP and 4PP were analyzed because of the prominent difference in the initial cell proliferation assays.

### Measurement of reactive oxygen species (ROS)

ROS production was evaluated similar to that described by the DCFDA Cellular ROS Detection Assay Kit (Abcam, Cambridge, UK), using 2′,7′-dichlorodihydrofluorescein diacetate (DCFDA) as a fluorescent probe. Cells were plated in a density of 3.0 × 10^4^ cells/well in a 96-well clear-bottom black plate. After treatments time (4 h and 24 h) cells were incubated with DCFDA (25 µM in PBS) in darkness for 45 min at 37 °C. After incubation, cells were washed and the fluorescence was measured (excitation/emission = 485/535 nm) using a Biotek Synergy H1 Hybrid Reader (Biotek, Winooski, USA). At the same time cell viability for the same treatments were done by MTT. The fluorescence intensity was calculated in relation to the correspondent cell viability after treatments.

### Water-soluble fraction characterization

#### Monosaccharide analysis

Samples of WSF from papaya were carboxyl-reduced with NaBD_4_ after activation with carbodiimide, as described by Kim and Carpita^71^ and modified by Carpita and McCann^72^. The sugar alditol acetates were prepared according to Gibeaut and Carpita^73^. Derivatives were separated by gas-liquid chromatography (GLC) on a 0.25-mm × 30-m column (SP-2330, Supelco, Bellefonte, PA). Temperature was held at 80 °C during injection, then ramped to 170 °C at 25 °C·min^−1^, and then to 240 °C at 5 °C·min^−1^, with a 10 min hold at the upper temperature. Helium flow was 1 mL min^−1^ with splitless injection. The electron impact mass spectrometry (EIMS) was performed with a Hewlett-Packard MSD at 70 eV with the temperature source at 250 °C. The proportion of 6,6-dideuteriogalactosyl was calculated using pairs of diagnostic fragments (*m/z* 187/189, 217/219 and 289/291) according to Kim and Carpita^71^.

#### Linkage analysis

For linkage analysis 1 mg of WSF was per-*O*-methylated according to Gibeaut and Carpita^73^. The partially methylated alditol acetates were separated on the same column as the alditol acetates. After a hold of 1 min at 80 °C during injection, the derivatives were separated in a temperature program of 160 °C to 210 °C at 2 °C·min^−1^, then to 240 °C at 5 °C·min^−1^, with a pause of 5 min at the upper temperature. All derivative structures were confirmed by electron-impact mass spectrometry^74^.

#### Homogeneity and molecular weight

Molecular mass distribution of the papaya WSF was analyzed by high performance size exclusion chromatography coupled to a refractive index detector (HPSEC-RID) using a 1250 Infinity system (Agilent, Santa Clara, CA). Samples were diluted with water (1 mg/mL), injected (25 µL) and separation was conducted through four PL aquagel-OH MIXED-M (300 × 7.5 mm, 8 μm) columns in tandem (Agilent, Santa Clara, CA). The eluent was 0.2 M NaNO_3_ at 35 °C with a flow of 0.6 mL·min^−1^. Molecular weights were estimated using dextran T-series (5, 25, 50, 80, 150 and 410 kDa; Fluka^TM^) as external standards.

#### Determination of Degree of O-Methyl Esterification

Lyophilized samples were used to determine the degree of *O*-methyl esterification using a Bruker Alpha Fourier Transform-Infrared (FTIR) spectrometer (Bruker Optic GmbH, Ettlingen, Germany), equipped with deuterated triglycine sulfate (DTGS) detector and a single bounce attenuated total reflectance (ATR) accessory (diamond crystal). FTIR–ATR spectra were obtained with 4 cm^−1^ resolution and a total of 50 scans were co-added. The spectra were analyzed with the GRAMS/AI (v. 9.1) package (Thermo Scientific). Methyl esterified and free uronic acids correspond to the band areas at 1749 cm^–1^ and 1630 cm^–1^, respectively. Pectins of known degrees of *O*-methyl esterification (Sigma) were analyzed and a standard curve was constructed according to Manrique and Lajolo^75^ to determine the degree of *O*-methyl esterification of papaya pectin.

#### Atomic force microscopy (AFM)

Aqueous samples (5 μL of concentration 2.5 μL/mL) were sonicated, drop deposited on freshly cleaved mica and dried over in vacuum at 30 °C for 20 min. The samples were maintained in desiccator until analysis. Topography images were obtained in a NX-10 Atomic Force Microscope (Park Systems, Suwon, South Korea) in an acrylic glove box with controlled temperature (around 22 °C) and humidity (around 3%). AFM imaging was acquired at tapping mode using a NCHR probe (NanoWorld) with a spring constant of 42 N/m and 320 kHz resonance frequency. The imaging was obtained with a scan speed of 0.5 Hz with a scanning resolution of 512 × 512 points. For each sample, at least 10 images were collected. Image measurements and automatic processing (plane subtraction and rows alignment) were performed using Gwyddion 2.47 software (http://gwyddion.net/).

#### Oligosaccharides qualitative analysis

Papaya pectin solutions (1 mg/mL) were analyzed using a PA1 pellicular anion-ex-change analytical (Dionex, 250 × 4 mm) column with its respective guard column in a ICS5000 + HPAEC-PAD System (Thermo-Dionex) with a gold working electrode and an Ag/AgCl reference electrode and an AS-AP autosampler to detect cell wall polysaccharides oligomers. NaOH 100 mM was used as eluent, with a 50 mM NaOAc gradient from 50 to 600 mM starting at 5 min and ending at 25 min. Column temperature was set in 30 °C and a cleaning step of 10 min of NaOH 50 mM containing 600 mM of NaOAc was added.

### Statistics

The results were expressed as the mean ± standard deviation (SD). Data were analyzed using GraphPad Prism version 6.0 software (GraphPad Software, San Diego, CA using one-way ANOVA with Tukey’s (to assess differences between all groups) or Dunnett’s (to assess differences between the control and two or more groups) post hoc tests. Values of *P* < 0.05 were considered as statistically significant.

## Electronic supplementary material

Supplementary Material
