# Supplementary material for: Ripening-induced chemical modifications of papaya pectin inhibit cancer cell proliferation
Source: Sci Rep. 2017 Nov 29;7:16564. doi: 10.1038/s41598-017-16709-3 (PMC5707353; doi:10.1038/s41598-017-16709-3)
Supplement: Supplementary file 1 — Supplementary Material [file 41598_2017_16709_MOESM1_ESM.pdf]

Supplementary information

Ripening-induced chemical modifications of papaya pectin inhibit cancer cell proliferation

Samira Bernardino Ramos do Prado[1], Gabrielle Fernandez Ferreira[1], Yosuke Harazono[2,3], Tânia Misuzu Shiga[1], Avraham Raz[2], Nicholas C. Carpita[4], João Paulo Fabi[1,5,6*]

[1]Department of Food Science and Experimental Nutrition, School of Pharmaceutical Sciences, University of São Paulo, São Paulo, SP, Brazil
[2]Departments of Oncology and Pathology, School of Medicine, Wayne State University, and Karmanos Cancer Institute, Detroit, MI, USA.
[3]Department of Maxillofacial Surgery, Tokyo Medical and Dental University, Bunkyo-ku, Tokyo 113–8510, Japan.
[4]Department of Botany & Plant Pathology, Purdue University, West Lafayette, IN, USA.
[5]Food and Nutrition Research Center (NAPAN), University of São Paulo, São Paulo, SP, Brazil.
[6]Food Research Center (FoRC), CEPID-FAPESP (Research, Innovation and Dissemination Centers, São Paulo Research Foundation), São Paulo, SP, Brazil.

*Correspondence: Departamento de Alimentos e Nutrição Experimental, FCF, Universidade de São Paulo, Av. Lineu Prestes 580, Bloco 14, CEP 05508-900, São Paulo – SP, Brazil. Tel. +55 11 30913647, email: jpfabi@usp.br

Table S1. Papaya water-soluble polysaccharides linkage analysis (PP: papaya pectin from different harvesting days – 1, 2 3 and 4).

Monomer	1PP	2PP	3PP	4PP
	Mol (%)			
t-Rha	0.8 ± 0.2	0.6 ± 0.3	0.7 ± 0.2	0.5 ± 0.1
2-Rha	0.8 ± 0.2	1.6 ± 0.3	2.0 ± 0.2	1.7 ± 0.1
2,4-Rha	0.1 ± 0.1	0.7 ± 0.2	1.1 ± 0.1	1.1 ± 0.1
t-Fuc	0.0 ± 0.0	0.0 ± 0.0	0.0 ± 0.0	0.0 ± 0.0
2,4-Fuc	0.1 ± 0.1	0.1 ± 0.1	0.1 ± 0.1	0.1 ± 0.1
t-Araf	2.1 ± 0.1	1.1 ± 0.2	0.6 ± 0.1	0.5 ± 0.1
t-Arap	0.9 ± 0.1	0.5 ± 0.2	0.8 ± 0.1	0.7 ± 0.1
2-Araf	0.1 ± 0.0	0.1 ± 0.1	0.1 ± 0.1	0.1 ± 0.0
3-Araf	0.7 ± 0.1	0.3 ± 0.1	0.4 ± 0.1	0.4 ± 0.1
5-Ara(f)	1.0 ± 0.1	0.5 ± 0.1	0.8 ± 0.1	0.7 ± 0.1
2,5-Ara(f)	0.0 ± 0.0	0.0 ± 0.0	0.0 ± 0.0	0.0 ± 0.0
3,5-Ara(f)	0.0 ± 0.0	0.0 ± 0.0	0.1 ± 0.1	0.0 ± 0.0
Ara(OAc)5	1.0 ± 0.2	0.9 ± 0.1	0.7 ± 0.1	0.6 ± 0.1
t-Xylp	0.7 ± 0.2	0.2 ± 0.1	0.4 ± 0.1	0.2 ± 0.1
2-Xyl	0.2 ± 0.1	0.2 ± 0.1	0.1 ± 0.1	0.1 ± 0.1
2,4-Xyl	0.1 ± 0.1	0.1 ± 0.1	0.1 ± 0.0	0.1 ± 0.0
4-Xyl	0.9 ± 0.1	0.8 ± 0.1	0.4 ± 0.1	0.5 ± 0.1
3,4-Xyl	0.1 ± 0.1	0.1 ± 0.1	0.1 ± 0.1	0.1 ± 0.1
Xyl(OAc)5	0.3 ± 0.1	0.5 ± 0.2	0.2 ± 0.1	0.2 ± 0.1
t-Man	0.0 ± 0.0	0.0 ± 0.0	0.0 ± 0.0	0.0 ± 0.0
4-Man	0.3 ± 0.1	0.3 ± 0.1	0.1 ± 0.1	0.3 ± 0.1
4,6-Man	0.0 ± 0.0	0.0 ± 0.0	0.0 ± 0.0	0.0 ± 0.0
Man(OAc)6	0.7 ± 0.1	0.7 ± 0.1	0.5 ± 0.1	1.0 ± 0.1
t-Gal	0.8 ± 0.1	1.0 ± 0.3	2.6 ± 0.6	1.8 ± 0.3
2-Gal	0.1 ± 0.1	0.2 ± 0.1	0.1 ± 0.1	0.1 ± 0.1
3-Gal	0.2 ± 0.1	0.1 ± 0.1	0.2 ± 0.1	0.3 ± 0.1
3,4-Gal	0.6 ± 0.2	0.5 ± 0.2	0.2 ± 0.1	0.1 ± 0.1
4-Gal	1.0 ± 0.1	0.8 ± 0.2	1.1 ± 0.2	0.3 ± 0.1
2,4-Gal	0.3 ± 0.1	0.8 ± 0.2	0.3 ± 0.1	0.4 ± 0.2
3,6-Gal	4.4 ± 0.1	2.7 ± 0.3	2.7 ± 0.2	1.9 ± 0.3
4,6-Gal	0.4 ± 0.1	0.4 ± 0.1	1.1 ± 0.4	0.4 ± 0.2
6-Gal	4.6 ± 0.4	1.9 ± 0.3	2.1 ± 0.2	2.2 ± 0.2
2,4,6-Gal	0.1 ± 0.1	0.4 ± 0.3	0.2 ± 0.1	0.4 ± 0.2
Gal(OAc)6	1.2 ± 0.5	2.3 ± 0.6	1.2 ± 0.3	1.0 ± 0.2
t-GalA	1.9 ± 0.4	2.6 ± 0.5	7.9 ± 2.8	4.2 ± 0.2
4-GalA	60.7 ± 2.4	64.3 ± 2.1	58.5 ± 3.5	68.5 ± 2.0
3,4-GalA	0.4 ± 0.2	0.5 ± 0.1	0.4 ± 0.2	0.3 ± 0.1
4,6 GalA	6.4 ± 1.3	8.0 ± 1.1	5.5 ± 0.6	5.0 ± 1.1
2,4 GalA	2.6 ± 1.1	5.1 ± 2.1	2.8 ± 0.7	1.8 ± 0.5
t-Glc	0.3 ± 0.1	0.1 ± 0.1	0.2 ± 0.2	0.1 ± 0.1
2-Glc	0.1 ± 0.1	0.1 ± 0.2	0.1 ± 0.2	0.1 ± 0.1
3-Glc	0.4 ± 0.1	0.1 ± 0.1	0.2 ± 0.1	0.2 ± 0.1
4-Glc	0.8 ± 0.1	1.1 ± 0.1	0.7 ± 0.1	0.6 ±0.1
3,4-Glc	0.3 ± 0.1	0.3 ± 0.3	0.9 ± 0.2	0.7 ± 0.1
4,6-Glc	0.1 ± 0.1	0.8 ± 0.1	0.1 ± 0.2	0.1 ± 0.1
Glc(OAc)6	0.5 ± 0.1	0.5 ± 0.1	0.6 ± 0.1	0.5 ± 0.1
t-GlcA	1.1 ± 0.3	1.8 ± 0.5	1.5 ± 0.3	1.8 ± 0.3

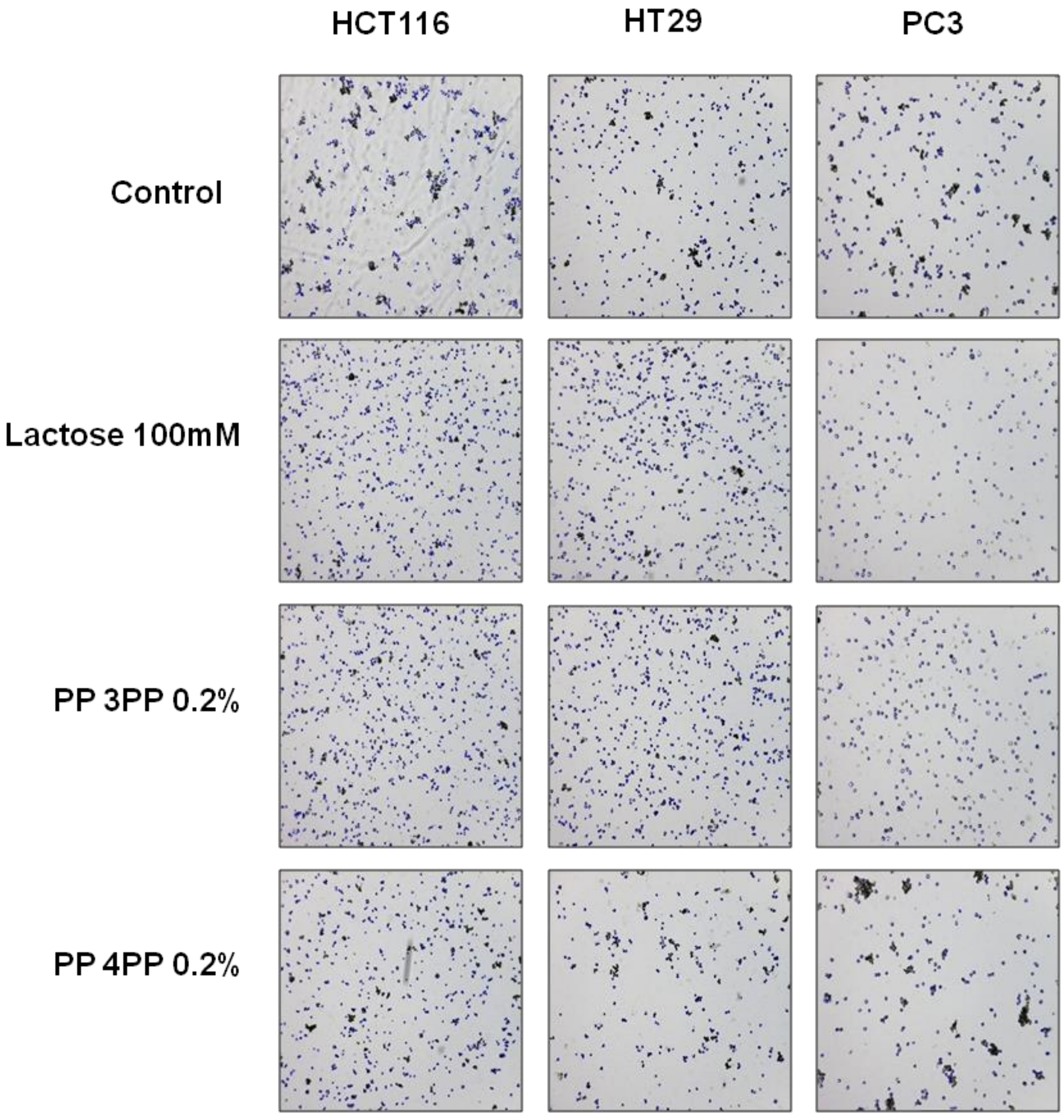

Figure S1. Homotypic aggregation. Cells were counted in an automatic cell counter and the blue circles were automatic generated by the machine software and is related to single or double cells.

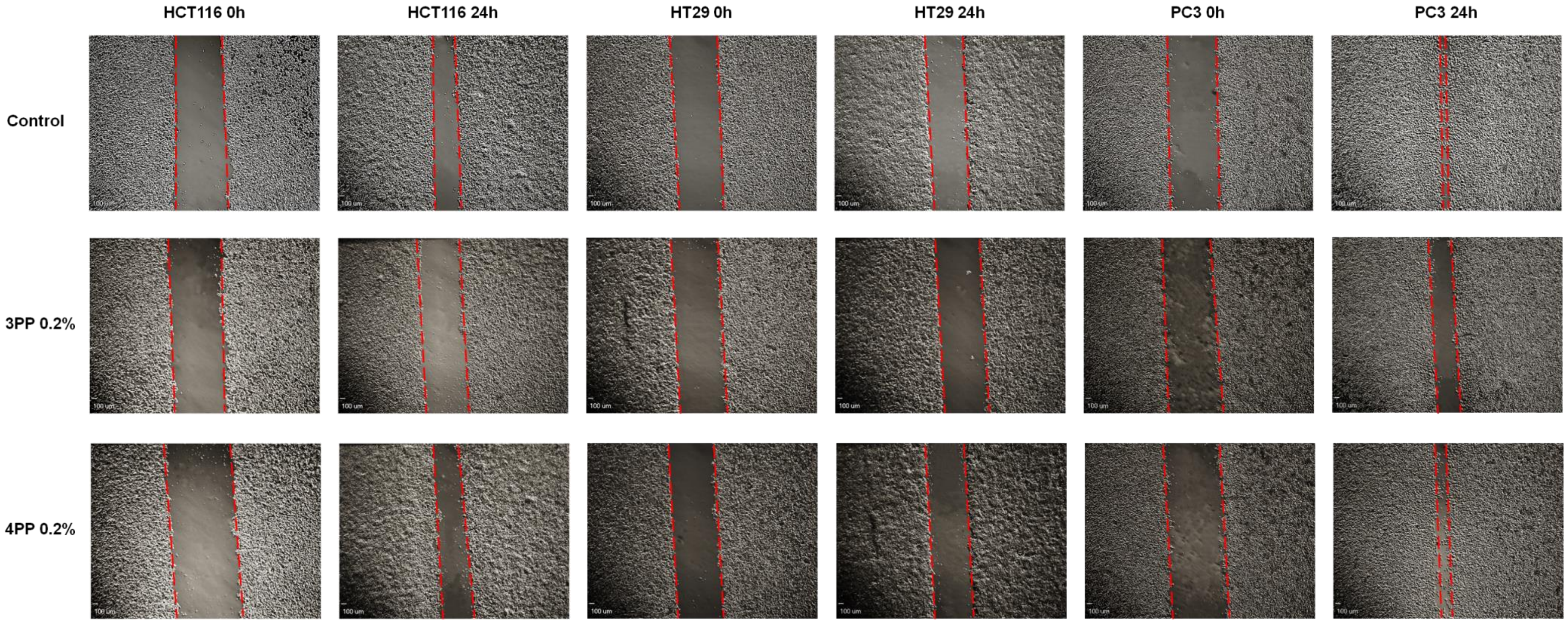

Figure S2. Migration assay based on wound healing assay. Photographs of cancer cell cultures in 0 h (after scratching) and after 24 h of treatment or not (control) indicating the wound cloasure. Scale bar: 100 µm.

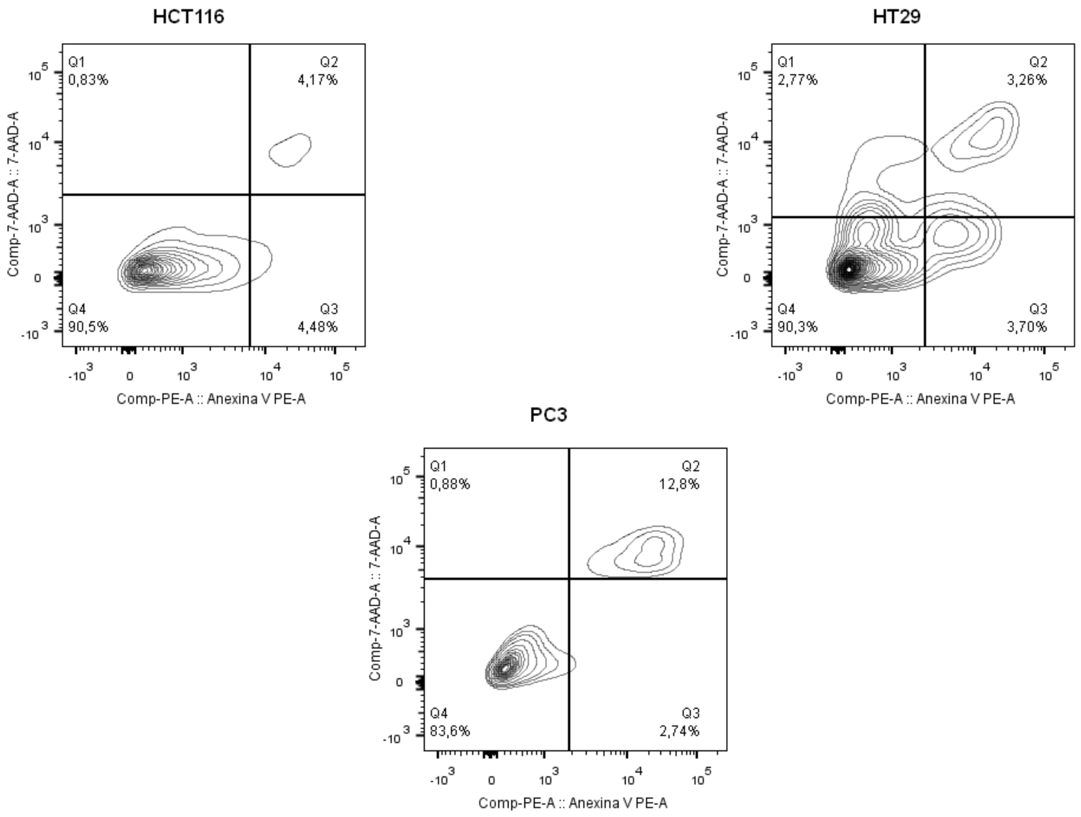

Figure S3. Effects of lactose in HCT116, HT29 and PC3 apoptosis by flow cytometry. Lactose did not induce cell death. Cells were treated with 100 mM of lactose for 24 h.

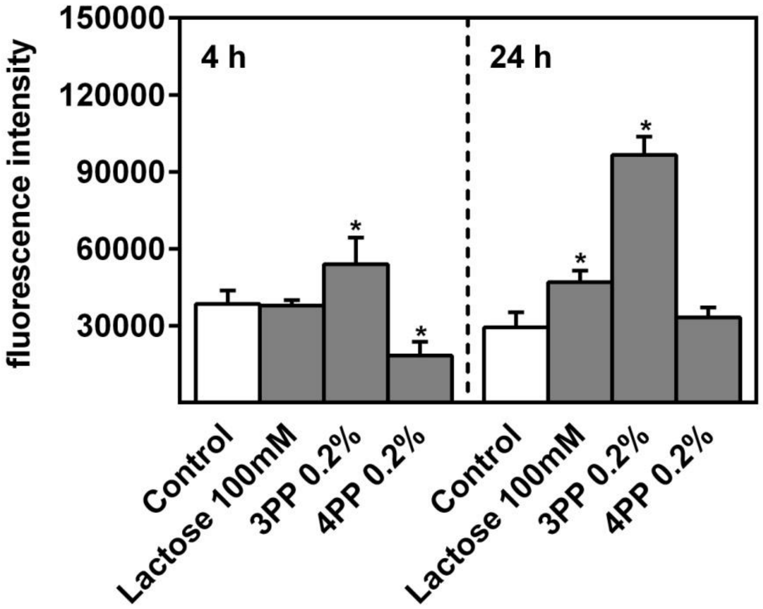

Figure S4. ROS detection on HCT116 cells measured by DCFDA. 3PP and not 4PP induced ROS accumulation. Cells were treated and after 4 h or 24 h cells were incubated with DCFDA for 45 min. Data was expressed as mean ± SD of fluorescence ratios for two independent experiments. **P* < 0.05 vs control, according to Dunnetts's test. PP: papaya pectin (water-soluble fraction).

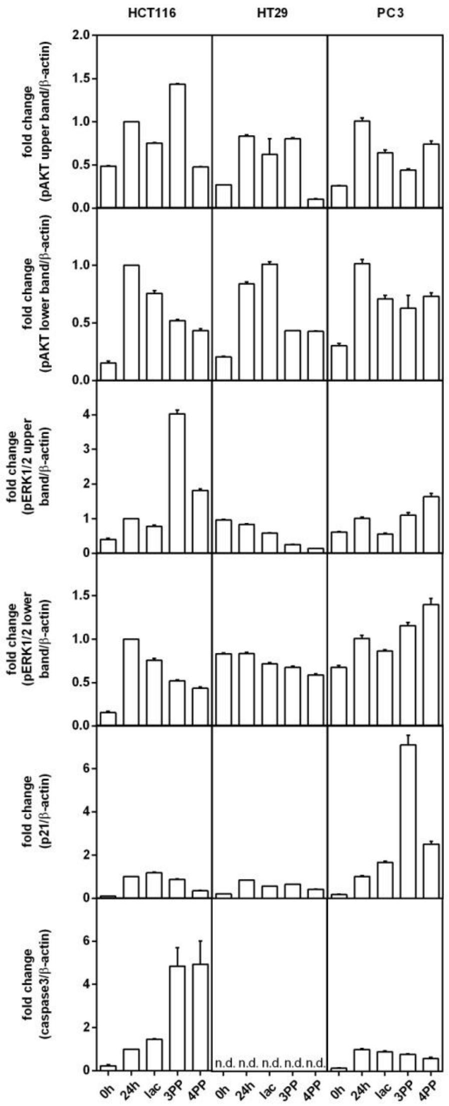

Figure S5. Relative density of bands from western blotting experiments. PP: papaya pectin (water-soluble fraction). Band intensities were separated in upper and lower bands for pAKT and pERK1/2 from two experiments.

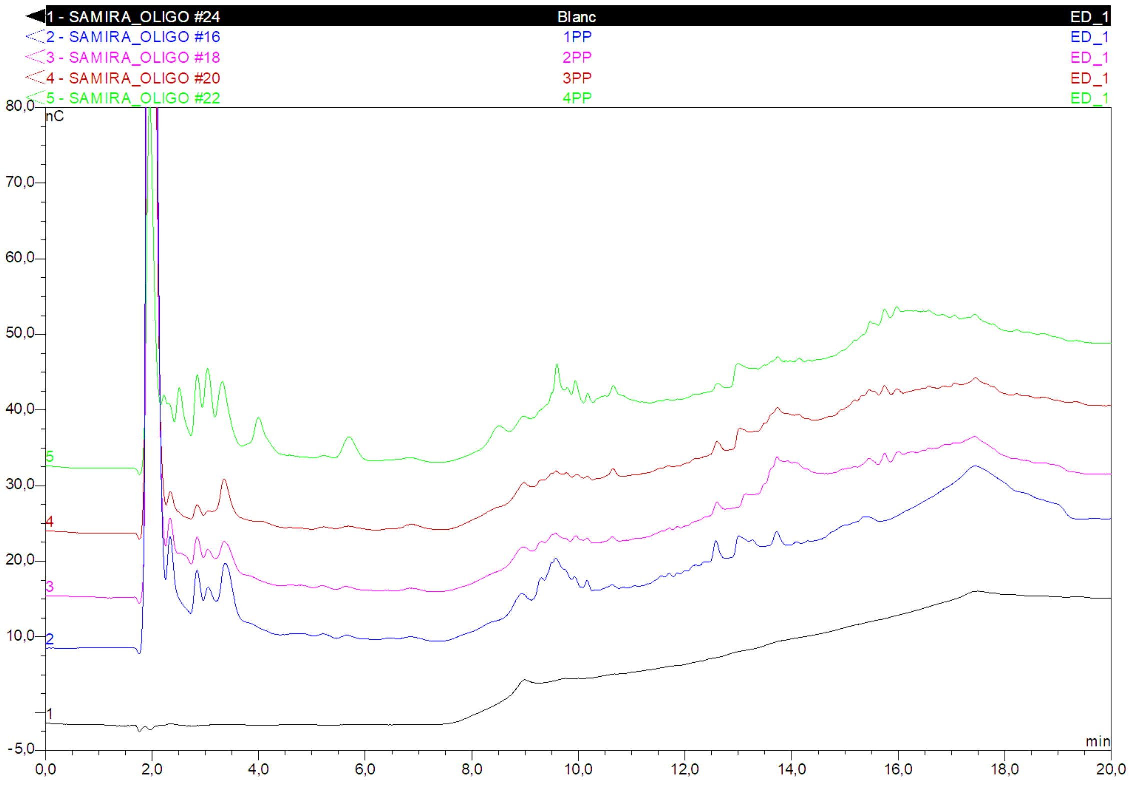

Figure S6. Qualitative analysis for oligosaccharide detection in papaya pectins. Black line: Blank. Blue line: 1PP. Purple line: 2PP. Red line: 3PP. Green line: 4PP.

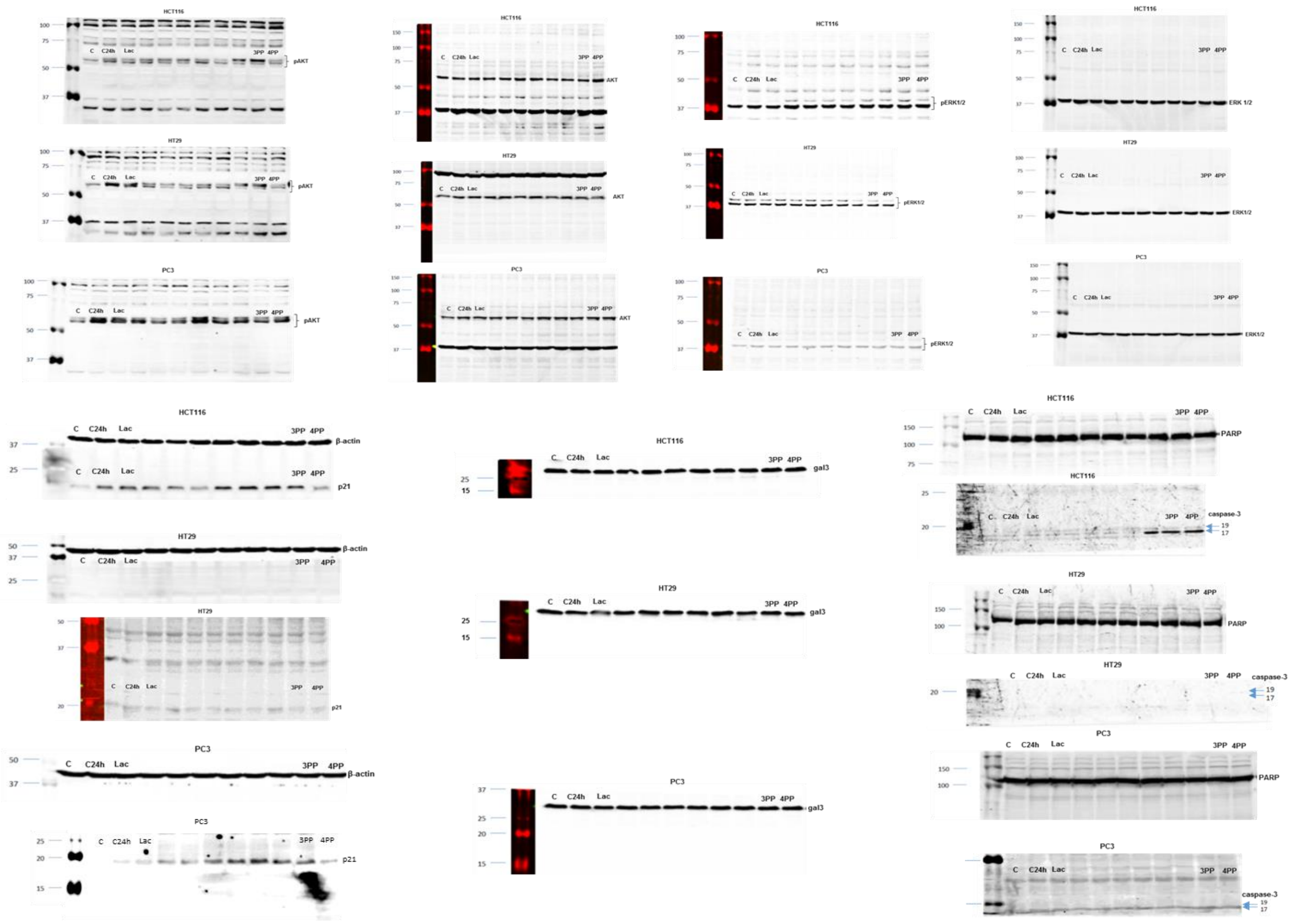

Figure S6. Representative western blotting from proteins analyzed in the article.
